# Atomic‐Scale Dynamics at the Interface of Doped Liquid Gallium: Contrasting Effects of Gallium Oxide and Vacuum

**DOI:** 10.1002/smsc.202500153

**Published:** 2025-05-15

**Authors:** Krista G. Steenbergen, Stephanie Lambie, Charlie Ruffman, Nicola Gaston

**Affiliations:** ^1^ MacDiarmid Institute for Advanced Materials and Nanotechnology Department of Physics University of Auckland Private Bag 92019 Auckland New Zealand; ^2^ Electronic Structure Theory Department Max Planck Institute for Solid State Research Heisenbergstraße 1 70569 Stuttgart Germany

**Keywords:** dopant dynamics, gallium, gallium oxide, interface phenomena, liquid metals, machine learning force fields, surface structuring

## Abstract

Liquid gallium exhibits a unique, geometrically structured surface that directly influences the diffusion and coalescence of metal solutes at its surface. The complex interplay between different chemical species and gallium's unusual interfacial properties remains poorly understood, yet it plays a crucial role in controlling dopant dynamics, with applications spanning catalysis, nanoscale fabrication, flexible electronics, and liquid metal batteries. Herein, large‐scale simulations with *ab* *initio*‐trained machine learning force fields reveal strikingly different interactions of Ag, Au, Bi, Li, Pt, and Sn with liquid gallium interfaces, including both liquid‐vacuum and liquid‐gallium oxide boundaries. For example, Bi dopants migrate strongly toward vacuum interfaces but are repelled by the oxide interface, while Au is repelled by both interfaces. The results have direct implications for applications involving doped liquid gallium systems, including optimizing Bi surface patterning in plasmonic and catalytic applications or the use of Li in liquid metal batteries. More broadly, these findings underscore the critical role of interfaces in modulating dopant dynamics, offering new pathways for tuning the properties and functionalities of liquid metal technologies.

## Graphical abstract

**GA:** Liquid gallium doped with Ag, Au, Bi, Li, Pt, and Sn exhibits strikingly different interfacial behavior at oxide versus vacuum interfaces. Discovered through large‐scale machine learning force field simulations, these findings reveal key dopant‐specific interactions at each interface which are critical for advancing liquid metal technologies.

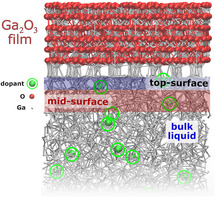

## 1 Introduction

Liquid metals (LMs) have recently attracted a remarkable amount of scientific and technological interest, sparked by their unique combination of metallic conductivity, fluidity, and reactivity.^[^ ^1^, ^2^, ^3^, ^4^, ^5^, ^6^ ^]^ These materials hold immense promise for a variety of applications, ranging from flexible electronics to catalysis and advanced coatings.^[^ ^1^, ^2^, ^3^, ^4^, ^5^, ^6^, ^7^, ^8^, ^9^, ^10^, ^11^, ^12^, ^13^, ^14^, ^15^, ^16^, ^17^ ^]^ LM systems are highly tunable, with differing dopant ratios, cooling rates, or oxygen exposure often leading to dramatic changes in experimental results.^[^ ^4^, ^18^, ^19^, ^20^ ^]^ This tunability and their inherent dynamic nature make them not only a fascinating topic for fundamental research but also a platform for advancing next‐generation technologies.^[^ ^3^, ^5^, ^11^, ^12^, ^15^, ^21^, ^22^, ^23^, ^24^ ^]^

While many dopants influence liquid metal properties in predictable ways, bismuth stands out as an exception. It is well‐known that bismuth exhibits anomalous behavior in liquid metal systems, often defying trends observed with other dopants. Studies have shown that even a small ratio of Bi can lower the surface tension in many liquid metals,^[^ ^25^ ^]^ induce unexpected segregation behaviors at interfaces,^[^ ^4^, ^5^, ^26^, ^27^ ^]^ and mo structural ordering within the liquid phase.^[^ ^28^ ^]^ These unique properties of bismuth as a dopant have been observed across a range of liquid metal environments, suggesting underlying mechanisms distinct from those governing more conventional dopant behavior.^[^ ^5^, ^27^, ^29^, ^30^ ^]^ What makes Bi unique from most other metal solutes, however, remains an open question.

Investigating such anomalous dopant behaviors, particularly in liquid metal solvents like gallium, requires simulations capable of capturing both bulk properties and interface dynamics. Yet, simulating gallium presents distinct challenges due to its structural complexity,^[^ ^31^, ^32^ ^]^ which persists into the liquid phase.^[^ ^33^ ^]^ Accurately capturing the properties of gallium (solid or liquid) requires first‐principles calculations, with the computational cost of electronic structure methods inherently limiting the size of possible simulations. However, studying surfaces and systems with a low dopant ratio necessitates (very) large simulation cells. The development of *ab initio*‐derived machine learning force field (MLFF) methods^[^ ^34^, ^35^, ^36^, ^37^, ^38^, ^39^ ^]^ has transformed materials science by enabling efficient, accurate simulations of materials at scales directly relevant to experiments.

**Figure 1:**
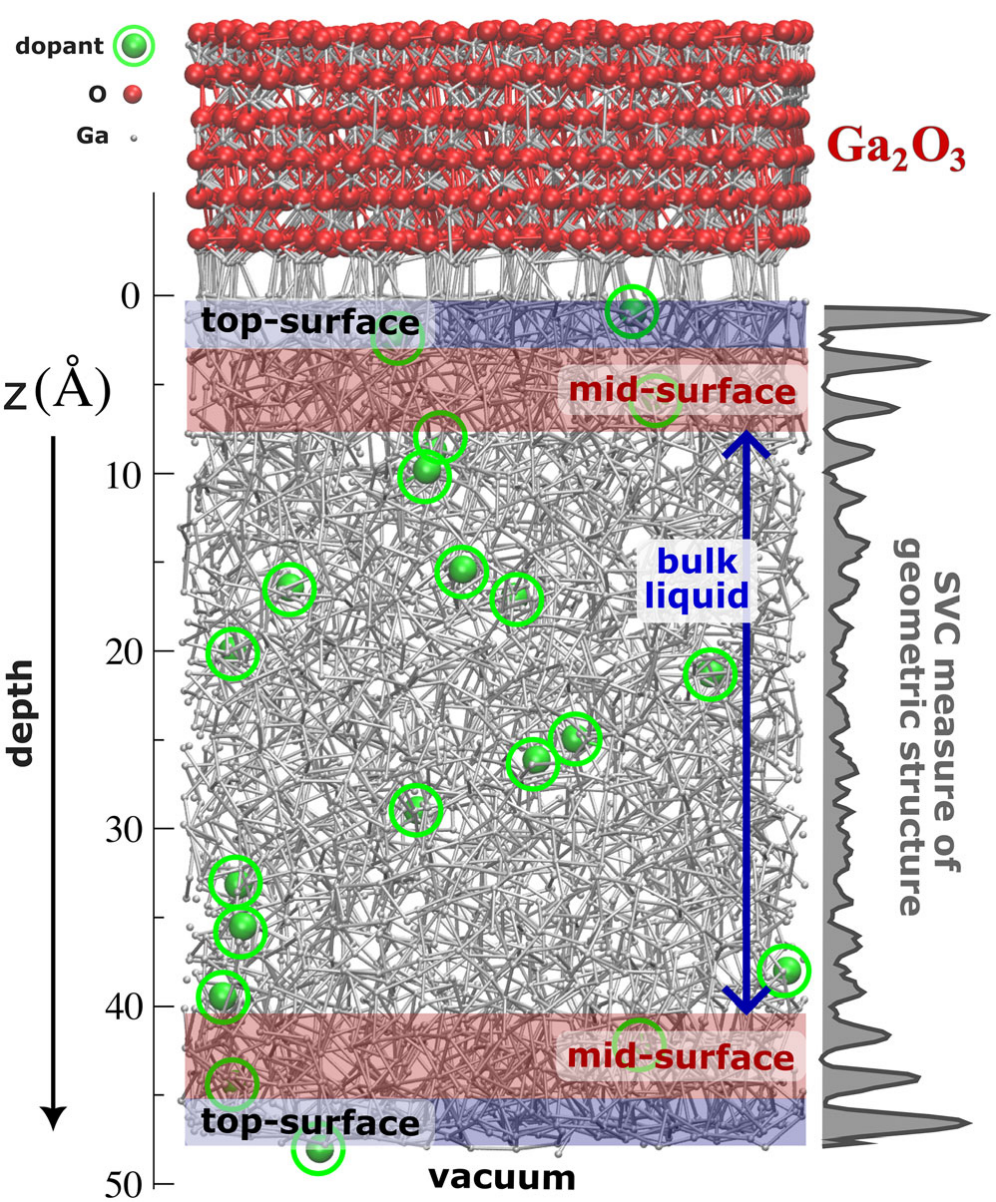
The MLFF simulation setup showing a snapshot of the unit cell. A 6‐layer α‐Ga_2_O_3_ film is interfaced with a liquid containing 3586 gallium atoms and 20 dopants, which are randomly placed and evenly distributed between the liquid‐vacuum and liquid‐oxide interfaces. The atomic sphere sizes are not physically representative, but are adjusted to highlight the dopants. We also show the variable we refer to as *depth*, z, which is a distance measured from the upper boundary of the gallium liquid (defined as the average *z*‐coordinate of the 20 liquid atoms closest to the oxide layer). The gray curve shown to the right of the simulation cell represents the SVC machine learning measure of local geometric structure, as described in our previous work.^[^ ^28^ ^]^ Geometric structuring is highest at the liquid‐oxide interface, slightly lower at the liquid‐vacuum interface, and remains elevated in two mid‐surface layers from both interfaces before falling to the bulk‐liquid level. Based on this SVC measure of local structure, three distinct regions can be identified in the gallium liquid: 1) the *top‐surface* region (<3 Å from either interface, highlighted in blue) with significant geometric structuring, 2) the *mid‐surface* region (>3 Å and <8.5 Å from either interface, highlighted in red), comprised of two additional surface layers exhibiting increased geometric order, and 3) a *bulk liquid* region (>8.5 Å from the interface) where no significant structuring is observed and the gallium liquid becomes fully disordered.

Our previous work validated the accuracy of *ab initio* molecular dynamics (AIMD) in simulating liquid gallium^[^ ^33^ ^]^ as well as the utility of *ab* *initio* trained MLFFs in uncovering a unique geometric structuring at the surface of liquid gallium.^[^ ^28^ ^]^ We were able to show that a support vector classification (SVC) machine learning analysis differentiates the degree of structuring and was used to identify three distinct regions in liquid gallium:^[^ ^28^ ^]^ 1) the *top‐surface* region with significant geometric structuring (<3 Å from either the oxide or vacuum interface), 2) the *mid‐surface* region, comprised of two additional surface layers with increased geometric order (>3 Å and <8.5 Å from an interface), and 3) a *bulk liquid* region where no significant ordering is observed and the gallium liquid becomes entirely disordered, as would be expected of a normal liquid (>8.5 Å from an interface). **Figure** **1** shows a sample simulation cell, exemplifying the three different liquid environments and the SVC measure quantifying geometric structure. Clearly, the geometric structure is most pronounced in the top‐surface layer nearest the oxideinterface, but the vacuum interface also exhibits significant structuring.

Here, we investigate the dynamic response of six dopants (Bi, Au, Pt, Sn, Li, and Ag) at gallium's liquid‐vacuum and liquid‐oxide interfaces using *ab initio*‐trained MLFFs. Each dopant has been experimentally studied in gallium alloys, with applications ranging from catalysis to nanostructure formation. These six were selected to span a range of sizes and chemical characteristics, while keeping the total number limited to enable deep statistical sampling across two interface types. Each simulation includes over 3,600 liquid atoms and a surface area exceeding 3,200 Å^2^, making these simulations nearly an order of magnitude larger than previous first‐principles studies.^[^ ^18^, ^20^, ^40^, ^41^ ^]^ Dopants are introduced at low concentrations (0.55 at% and 0.055 at%) with systematically varied initial depths (Figure 1), allowing for robust statistical analysis across 30 independent simulations per dopant. This combination of large‐scale modeling and statistical rigor provides new insight into dopant behavior at liquid metal interfaces, expanding upon our previous work in liquid metal characterization^[^ ^4^, ^14^, ^18^, ^19^, ^20^, ^28^, ^33^, ^41^, ^42^ ^]^ and catalysis.^[^ ^13^, ^43^, ^44^ ^]^ Among the six dopants studied, we find distinct interfacial preferences and mobility trends that defy simple size‐ or charge‐based explanations. These behaviors reveal the nuanced and element‐specific interplay between dopants and interfacial environments in liquid gallium.

## 2 Results

Simulation details are provided at the end of the main text and in Sections S1 and S2, Supporting Information. In brief, molecular dynamics (MD) simulations are performed at 450 K for 200 ps with a 1 fs timestep, incorporating periodic boundaries in two dimensions and vacuum in the third. An α‐Ga_2_O_3_ thin film is added to one liquid interface. The main simulations include 20 dopant atoms (0.55 at%) that are initially placed at evenly spaced depths between the two interfaces. We also include control simulations with a lower dopant ratio (0.055 at%) to examine concentration‐dependent effects, with dopants placed in bulk, mid‐surface, and top‐surface regions near either the oxide or vacuum interface. Our MLFFs are trained and validated (Section S1.2, Supporting Information) for low dopant concentrations where atoms remain well‐dispersed, ensuring accurate modeling of local environments without the need to capture dopant clustering effects.

**Figure 2:**
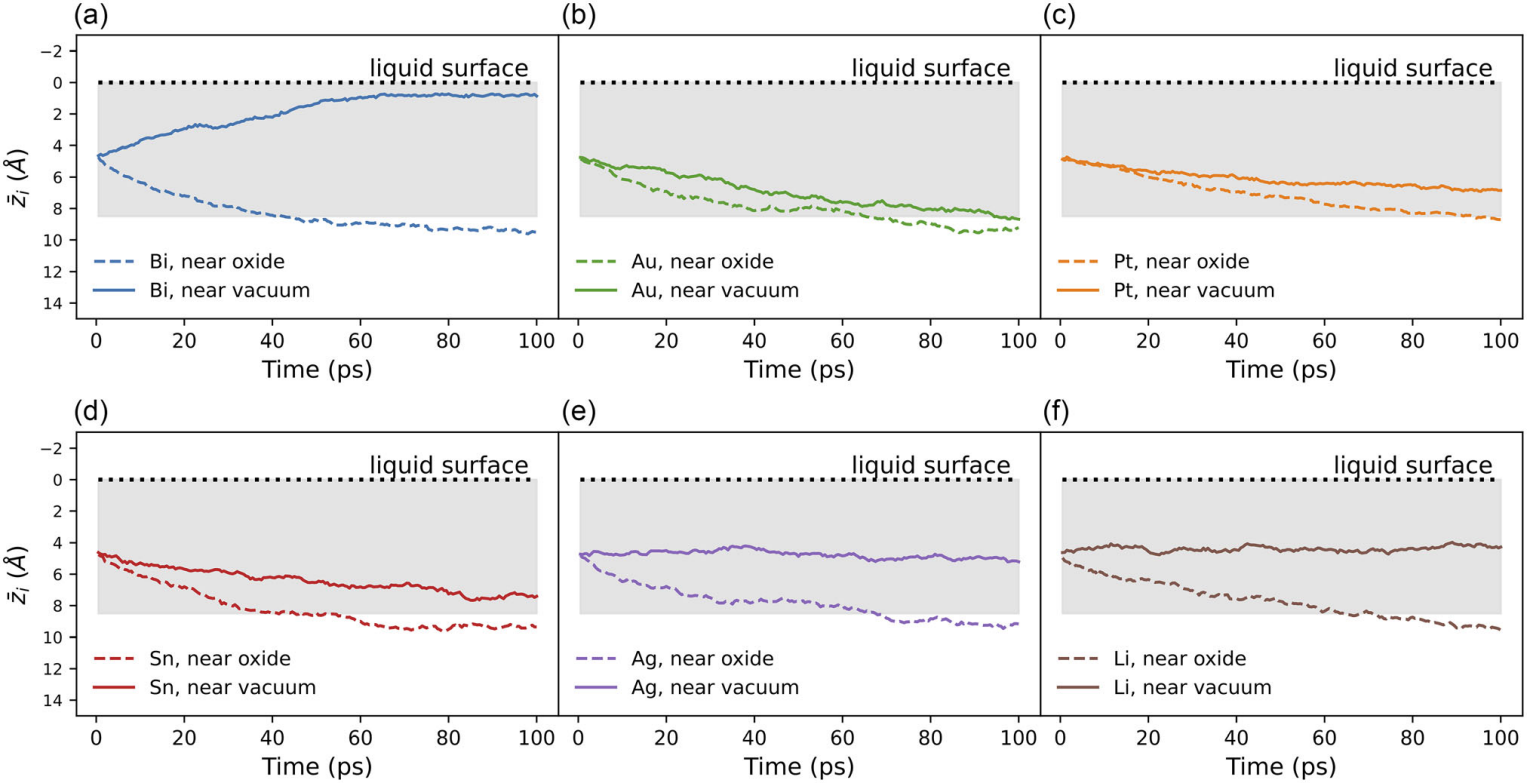
The $\bar{z}(t)$ results for the mid‐surface seeded dopant of the lower dopant (0.055 at%) simulations: (dashed lines) the mid‐surface dopant is initialized near the oxide interface; and (solid lines) the mid‐surface dopant is initialized near a vacuum interface. The horizontal dotted black line indicates the position of the gallium liquid surface nearest the oxide or vacuum. The gray shaded region denotes the cotop‐ and mid‐surface liquid regions within 8.5 Å of the liquid surface. Panels a–f) show results for Bi, Au, Pt, Sn, Ag and Li, respectively.

Dopant dynamics are quantified via diffusion coefficients and depth‐averaged traces, ${\bar{z}}_{i}(t)$, extracted throughout the simulations (detailed in Section S2, Supporting Information). Each dopant displayed different dynamics within each of the three liquid gallium regions (bulk, mid‐surface, and top‐surface), as shown in Figure 1. Results are grouped according to each dopant's response to the liquid's mid‐surface region (**Figure** **2**), as this provides the clearest distinction in dopant dynamics.

### 2.1 Bismuth

**Figure 3:**
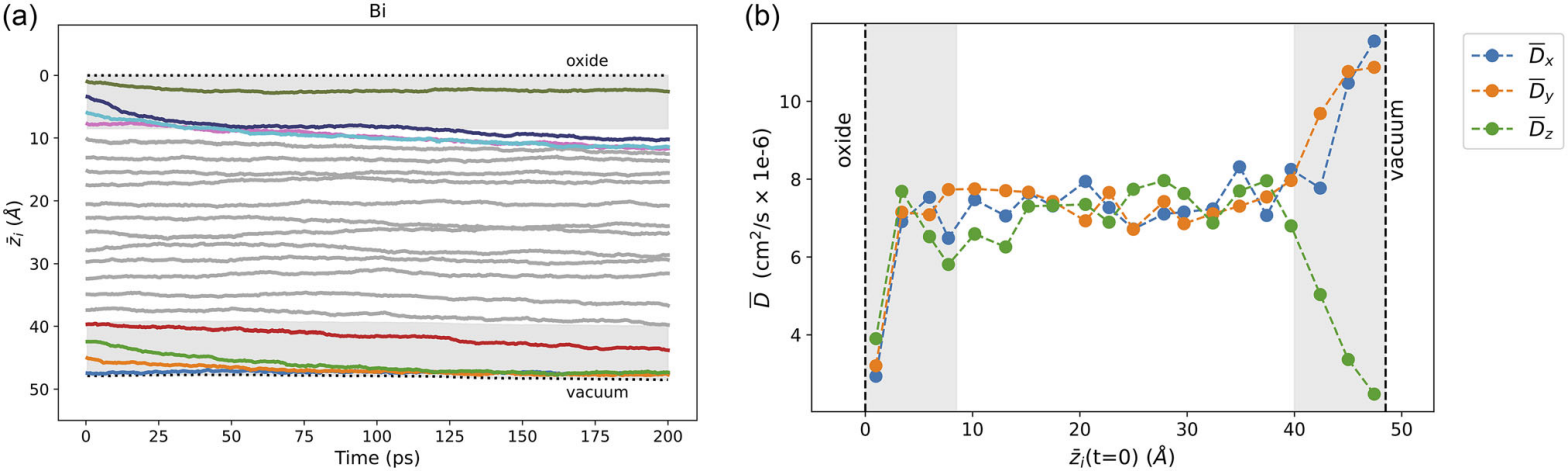
a) The average depth as a function of MD simulation time, $\bar{z}(t)$, is shown for each of the 20 Bi dopants. For each dopant, ${\bar{z}}_{i}(t)$ is averaged over 30 simulations (Section S2, Eq. 1) to represent the statistical behavior of the system. Colored lines indicate dopants that were initially within 8.5 Å of either interface; all others are shown in gray. Dashed horizontal lines mark the instantaneous liquid surface positions. b) Average directional diffusion coefficients (${\bar{D}}_{x}$, ${\bar{D}}_{y}$, and ${\bar{D}}_{z}$) for each Bi dopant as a function of its average initial *z*‐position, ${\bar{z}}_{i}(t=0)$. Diffusion is calculated in the Cartesian *x*, *y*, and *z* directions (see Section S2.1) and averaged over 30 simulations. The gray shaded regions in both (a) and (b) denote the top‐ and mid‐surface liquid regions within 8.5 Å of the liquid surface at both interfaces.

The dynamic behavior of bismuth is both intriguing and entirely distinctive from the other dopants. Figure 2a illustrates this for the 0.055 at% simulations, while the ${\bar{z}}_{i}(t)$ result for the 0.55 at% simulations (**Figure** **3**a) shows Bi dynamics in all three liquid regions. For all Bi dopants initialized within 8.5 Å of an interface, the dynamics differ markedly between the oxide and vacuum boundaries. Note that within 8.5 Å of an interface, the gallium liquid exhibits pronounced surface structuring (SVC to the right of Figure 1), beyond which the liquid becomes fully disordered, as expected of a typical bulk liquid.^[^ ^28^ ^]^

Near the oxide interface, dopants seeded in the mid‐ or top‐surface layers (top four lines in Figure 3a) are repelled, as indicated by the negative slope of $\bar{z}(t)$. The dopant initially placed within 3 Å of the oxide (cyan) appears less strongly repelled, but this reflects an averaging of two distinct behaviors: in 20 out of 30 simulations, that top‐seeded dopant becomes trapped at the interface, while in the remaining 10, it escapes the top‐surface layer within 25 ps and rapidly diffuses into the bulk liquid region.

In contrast, dopants seeded 3–8.5 Å from the vacuum interface (bottom‐most red, green, and orange lines) migrate swiftly toward that surface. The purple line in Figure 3a shows a dopant atom just outside this threshold, which also exhibits surface‐directed motion, albeit with a smaller slope. The single dopant initialized <3 Å from the vacuum interface (blue, $\bar{z}\approx$ 48 Å) remains confined to that top‐surface layer. When four simulations are extended to 0.5 ns, additional dopants initially seeded in the bulk region also migrate toward the vacuum, with some even being ejected (Figure S6c, Supporting Information). In contrast, no bismuth atoms near the oxide systematically migrate toward that interface over the entire 0.5 ns.

A look at the diffusion behavior of the 20 bismuth dopants relative to each interface is also intriguing. Figure 3b shows the average directional diffusion calculated in the three Cartesian directions as a function of the average initial *z*‐seeded position, ${\bar{z}}_{i}(t=0)$. Diffusion is calculated by the slope of the mean squared displacement, as detailed in Section S2.1. In the mid‐surface region near the vacuum‐interface (${\bar{z}}_{i}(t=0)>$ 40 Å), the lateral diffusion is ≈1.5 times that of the bulk diffusion rate, while the *z*‐diffusion is diminished. This means that while the bismuth dopant becomes trapped within the structured surface layers (low *z*‐diffusion), the mobility lateral to the surface is actually enhanced. Near the oxide interface, bismuth diffusion is diminished in all directions equally, but only for the top‐seeded atom ($\bar{z}(t=0)$ < 3 Å).

These MLFF results complement classical MD simulations that used an empirical force field to model liquid BiGa of comparable system size but with a higher dopant ratio.^[^ ^4^ ^]^ Similar to our findings, those simulations found that Bi dopants migrate toward the vacuum interface and experience reduced lateral diffusion near the oxide interface. However, they observed bulk‐like lateral diffusion at the vacuum interface (here, it increased) and Bi migration toward the oxide interface (here, Bi is repelled from the oxide). These discrepancies are likely attributable to differences in simulation parameters, including a different choice for the oxide film crystal structure (*β*‐Ga_2_O_3_), a lower simulation temperature (305 K), and a higher dopant ratio (1%).

These results also align with our previous AIMD study of bismuth dopants in small liquid gallium nanoparticles.^[^ ^4^, ^20^ ^]^ In that study, the bismuth dopants consistently migrated toward the nanoparticle surface (vacuum interface) or remained within the same radial layer as initially seeded. Due to the computational limitations of AIMD, this small nanoparticle model was required to capture surface effects. In contrast, the MLFF approach employed here enables *ab initio* accuracy for simulations of larger systems with extended, planar interfaces and a more substantial bulk region, thus allowing for a more comprehensive assessment of dopant behavior across different regions of the liquid gallium. Additionally, while the pure AIMD methodology was limited to vacuum interfaces, the MLFF approach allows us to investigate the effects of the oxide interface while maintaining first‐principles accuracy, overcoming the trade‐off between system size and simulation fidelity.

By bridging the gap between small‐scale AIMD simulations and large‐scale classical simulations, the present MLFF approach captures key structural nuances while maintaining computational efficiency. On this basis, we propose a clarified framework for interpreting the experimental findings in ref. [4]. The experimental results showed that when oxidation was negligible, the surface was Bi‐enriched but did not form patterns. In systems where a gallium oxide film was present, the oxide wrinkled upon cooling.^[^ ^4^ ^]^ Our $\bar{z}(t)$ and directional diffusion results (Figure 3a,b) suggest that the interplay between oxide wrinkling and Bi segregation at the liquid surface may be a critical factor in driving pattern formation. Applying our findings to the experimental observations, we propose that oxide wrinkling leads to partial detachment of the oxide film from the liquid, exposing regions of vacuum interface that strongly attract Bi dopants (Figure 2a, 3a and S6, Supporting Information). At these vacuum‐exposed regions, Bi remains largely confined to the interface due to low *z*‐diffusion, while exhibiting significantly enhanced lateral mobility (Figure 3b).

Conversely, regions where the oxide remains attached to the liquid surface tend to repel Bi (Figure 2a and 3a), which could account for the experimentally observed bismuth‐poor regions. Some Bi dopants may also migrate from the surface layer and become transiently trapped between the oxide and the liquid. These trapped dopants could, in turn, contribute to further oxide detachment, reinforcing a dynamic feedback loop between oxide wrinkling and Bi surface behavior. Further support for this mechanism comes from the observation that surface‐enriched Bi regions exhibited greater ordering when the liquid surface was mechanically flattened, suggesting that patterned oxide detachment, rather than a purely liquid‐phase phenomenon, could play a key role in driving the observed surface structures.

### 2.2 Gold, Platinum, and Tin

Gold, tin, and platinum also exhibit intriguingly strong responses to the gallium liquid mid‐surface layers, although the migration at the vacuum is opposite that of bismuth. As shown in Figure 2b–d, Au, Pt, and Sn mid‐surface dopants exhibit similar *z*‐dynamics in response to both the oxide and vacuum interfaces. All three dopant types move quickly from the structured mid‐surface region to the geometrically unstructured bulk‐liquid region which begins at 8.5 Å.

**Figure 4:**
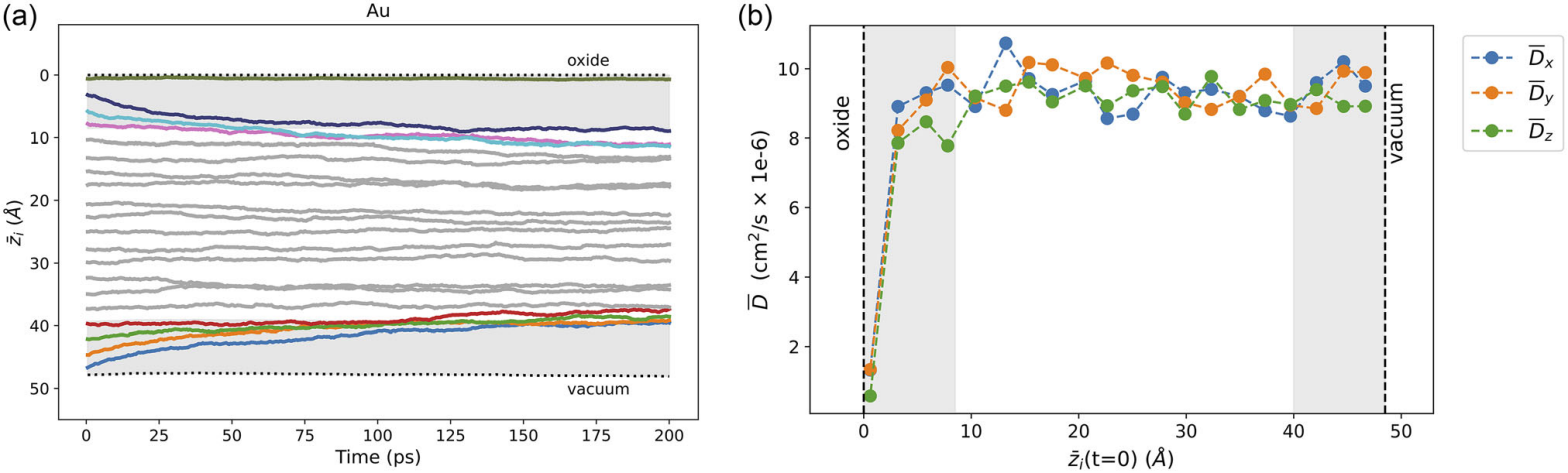
a) The average depth as a function of MD simulation time, $\bar{z}(t)$, is shown for each of the 20 Au dopants. For each dopant, ${\bar{z}}_{i}(t)$ is averaged over 30 simulations (Section S2, Eq. 1) to represent the statistical behavior of the system. Colored lines indicate dopants that were initially within 8.5 Å of either interface; all others are shown in gray. Dashed horizontal lines mark the instantaneous liquid surface positions. b) Average directional diffusion coefficients (${\bar{D}}_{x}$, ${\bar{D}}_{y}$, and ${\bar{D}}_{z}$) for each Au dopant as a function of its average initial *z*‐position, ${\bar{z}}_{i}(t=0)$. Diffusion is calculated in the Cartesian *x*, *y*, and *z* directions (see Section S2.1) and averaged over 30 simulations. The gray shaded regions in both (a) and (b) denote the top‐ and mid‐surface liquid regions within 8.5 Å of the liquid surface at both interfaces.

This dynamic behavior is further supported by the $\bar{z}(t)$ results for the 20‐dopant simulations of Au, Pt, and Sn, as illustrated in **Figure** **4** for Au (similar results are found for Pt and Sn, in Figures S5a and S5b, Supporting Information, respectively). All atoms in the mid‐surface layers (3–8.5 Å from an interface) move systematically away from the interfaces, both oxide and vacuum. These plots also clearly show that the Au, Pt, and Sn dopants prefer the bulk liquid environment to the ordered liquid surface layers, as the *z*‐migration away from the interface slows once the atoms are at least 8.5 Å from either interface, in the region of the true bulk liquid.^[^ ^28^ ^]^

The atoms seeded in the top‐surface layers are exceptions. For the top‐surface atom nearest the oxide (cyan line), the atom is not trapped between the surface and oxide film, but remains in the top‐surface layer within 1–2 Å of the liquid surface for the duration of the simulation. For Au and Pt, the top‐surface dopant seeded near the vacuum surface is repelled from the vacuum interface. For Sn, however, the top‐surface dopant near the vacuum becomes trapped in the top layer for the entirety of the simulation (Figures S7d and S5b, Supporting Information). This trapping could explain Sn migration to the surface in our previous 6‐layer Ga‐Sn slab models (without oxide), as the finite thickness of the model prevented the formation of a true bulk liquid region.^[^ ^42^ ^]^

AIMD simulations of GaPt slab systems have also documented Pt migration away from the surface.^[^ ^12^ ^]^ However, previous computational studies have demonstrated subsurface platinum enrichment in both NPs and in surfaces.^[^ ^14^, ^40^ ^]^ All three studies use a larger dopant ratio as well as considerably smaller unit cell sizes, necessitated by computational restraints to run full AIMD simulations. With the limited simulation sizes, the true bulk liquid environment (>8.5 Å from an interface) is never achieved, thereby limiting the extent of the observable dopant response to an interface.

Our results for Sn align well with an experimental study on GaSn systems, which found that Sn exhibits a preference for migrating away from the surface in the liquid phase.^[^ ^45^ ^]^ In the solid alloy, the surface Sn content was lower than the stoichiometric ratio; however, this depletion became more pronounced upon melting. While the experiment indicates a tendency for Sn to migrate away, it does not show complete surface depletion, suggesting that some Sn remains at the surface–consistent with our observation that top‐surface‐seeded Sn dopants stay at the vacuum or oxide interface, while others migrate away.

**Figure 5:**
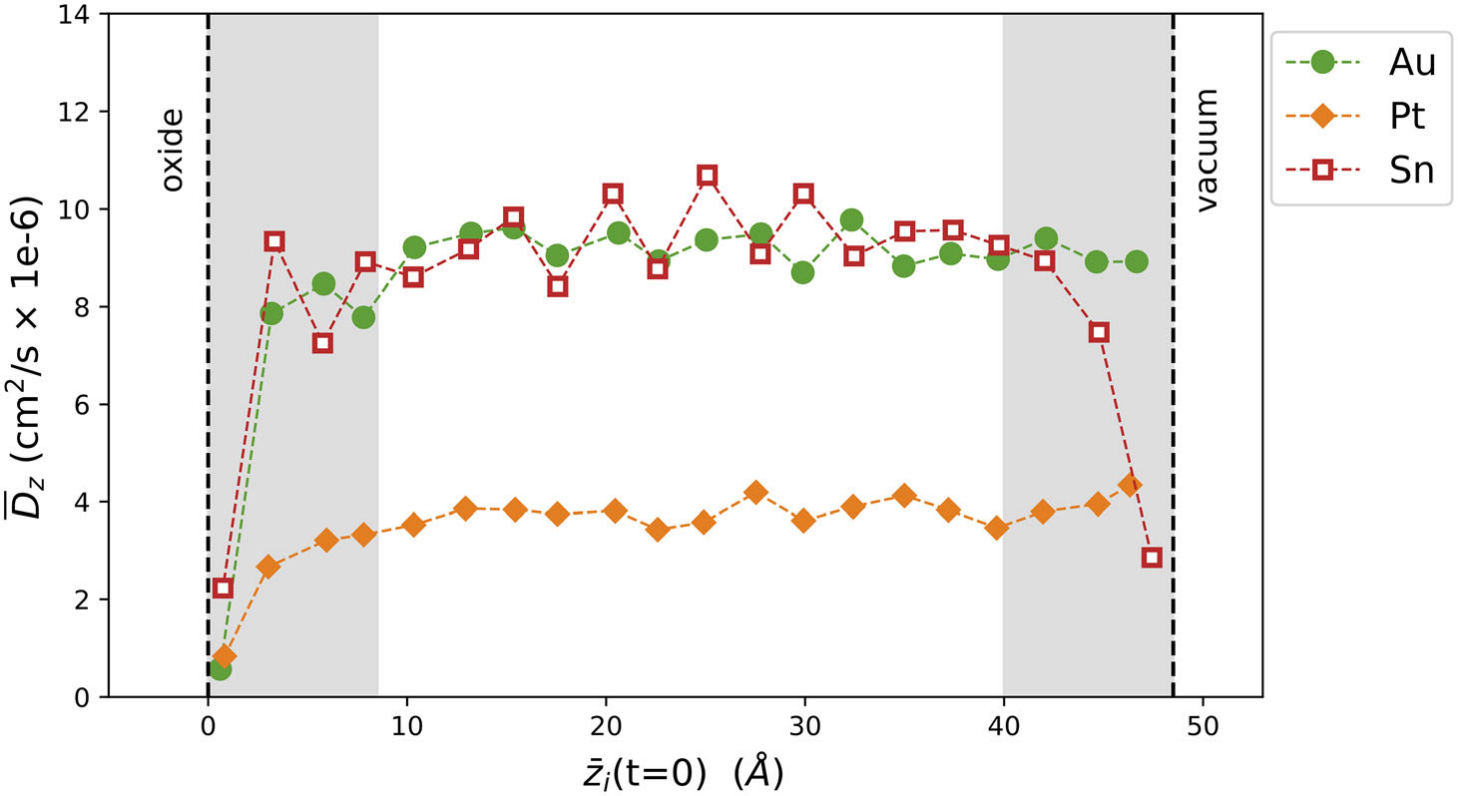
For the Au, Pt, and Sn simulations, ${\bar{D}}_{z}$ is given for each of the 20 dopants as a function of the average initial *z*‐position, ${\bar{z}}_{i}(t=0)$. The directional diffusion is calculated as described in Section S2.1, then averaged over all 30 simulations. For reference, dashed vertical lines show the approximate locations of the liquid surface nearest the indicated interface. The gray‐shaded regions denote the top‐ and mid‐surface liquid regions within 8.5 Å of the liquid surface at both interfaces.

A comparison of the *z*‐diffusion as a function of the average initial *z*‐position $\left(\bar{z}(t=0)\right)$ further clarifies these results, as shown in **Figure** **5**. The low *z*‐diffusion for the dopant nearest the oxide interface arises due to that atom's inclination to stay within that top surface layer. In fact, the *x* and *y* diffusion are equally low near that interface (Figure S8a, Supporting Information). For the top‐surface atom at the vacuum interface, Au and Pt have *z*‐mobility that is the same as the bulk liquid dopants; however, Sn exhibits low *z*‐mobility at the vacuum interface, as the dopant becomes trapped in that top‐surface layer.

Platinum exhibits strikingly low mobility for 20 dopant atoms throughout the surface and bulk liquid regimes, which is relatively unmodified by proximity to an interface (Figures S9a and S9b, Supporting Information) or by diffusion directionality (Figure S8b, Supporting Information). This is unsurprising due to the known strength of the Ga‐Pt interaction (Figure S11, Supporting Information),^[^ ^12^ ^]^ which effectively traps the Pt in the liquid gallium matrix.

### 2.3 Silver and Lithium

**Figure 6:**
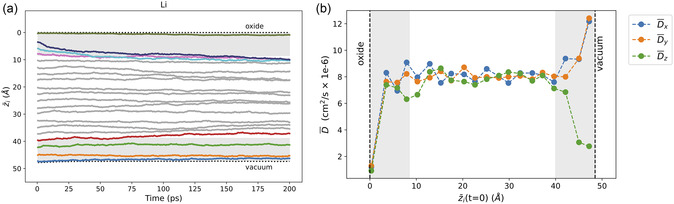
a) The average depth as a function of MD simulation time $\left(\bar{z}(t)\right)$ is shown for each of the 20 Li dopants. For each dopant, ${\bar{z}}_{i}(t)$ is averaged over 30 simulations (Section S2, Eq. 1) to represent the statistical behavior of the system. Colored lines indicate dopants that were initially within 8.5 Å of either interface; all others are shown in gray. Dashed horizontal lines mark the instantaneous liquid surface positions. b) Average directional diffusion coefficients (${\bar{D}}_{x}$, ${\bar{D}}_{y}$, and ${\bar{D}}_{z}$) for each Li dopant as a function of its average initial *z*‐position, ${\bar{z}}_{i}(t=0)$. Diffusion is calculated in the Cartesian *x*, *y*, and *z* directions (see Section S2.1) and averaged over 30 simulations. The gray‐shaded regions in both (a) and (b) denote the top‐ and mid‐surface liquid regions within 8.5 Å of the liquid surface at both interfaces.

As shown in Figure 2e,f, the mid‐surface‐seeded Ag and Li dopants appear unaffected by the geometric structure in the mid‐surface layers near the vacuum interface. The extra structuring introduced by the oxide interface, however, drives both dopants from the mid‐surface into the bulk liquid regime. This is confirmed by the 20‐dopant analysis, as shown in **Figure** **6**a and S5c, Supporting Information for Li and Ag, respectively. Both Li and Ag dopants seeded within 8.5 Å of the vacuum interface remain there for the entire 200 ps. The dopants from 3 to 8.5 Å of the oxide interface are repelled. However, the dopant seeded nearest to the oxide interface becomes trapped by that highly structured top‐surface layer.

These results align perfectly with previous AIMD results,^[^ ^18^ ^]^ from a study investigating the solidification behavior of GaAg systems with 0.1 at% Ag, which combined experiments and MD simulations to reveal how surface conditions influence formation of bifurcation and inverse bifurcation surface Ag patterns. Both their AIMD simulations and our findings demonstrate that Ag moves away from the top‐surface region of the Ga liquid. Their study was on a slab of only 16 Å, eliminating their ability to investigate Ag trends in the mid‐surface or bulk liquid regions (i.e., at 16 Å total, the bulk liquid region never materializes).

Directional diffusion results for Li and Ag are given in Figures 6b and S8c, Supporting Information, respectively. Diffusion is low in all three Cartesian directions at the oxide interface. At the vacuum interface, however, only *z* diffusion is low, while *x* and *y* diffusion in the mid‐surface layers is elevated. The lateral spreading behavior at the vacuum interface is very similar to that of Bi (Figure 3b). A key distinction for Bi, however, is that the dopants migrated strongly toward the vacuum interface (even the bulk liquid region), whereas Ag and Li remain in the mid‐surface layers without diffusing either toward or away from the vacuum interface.

## 3 Discussion

Our results suggest that Bi's divergent response to different interfaces may contribute to its experimentally observed surface enrichment.^[^ ^4^ ^]^ However, we have yet to explore *why* bismuth's dynamic behavior differs so dramatically from other dopants, such as gold. Examining the pair distribution function, *g*(*r*), for bulk liquid interactions given in Figure S12a, Supporting Information, we find that Bi—Ga interactions exhibit an elongated first and second nearest‐neighbor distance (3.1 and 5.8 Å) relative to pure Ga (2.8 and 5.3 Å). Taking these peaks to be an indication of the effective size of the dopant atom within the gallium matrix, this implies that the bismuth atom is simply too large for the bulk liquid and might be inclined to move toward the vacuum where shifting the surrounding gallium matrix would be less energetically costly (i.e., in the top‐surface layer near the vacuum, only 1/2 the bonds need to be elongated). We do not calculate explicit segregation energies, as the small energy differences involved would be overwhelmed by the large intrinsic fluctuations of the liquid gallium matrix at the low dopant concentrations studied (0.055% and 0.55%). Instead, we use dynamic trends, such as ${\bar{z}}_{i}(t)$ trajectories and diffusivity, as proxies for interfacial preference, since these persistent behaviors clearly reflect an underlying thermodynamic driving force despite the noisy energetic landscape. In contrast, although the oxide atoms are not constrained in the simulation and vibrate thermally, the solid nature of the oxide naturally limits atomic motion at the interface. Our previous work demonstrated that the oxide film causes a significant contraction of the liquid Ga—Ga first coordination distance, with a change to 2.7 Å in the top‐surface region, as well as an enhanced structuring in that liquid layer.^[^ ^28^ ^]^ This contraction would make the liquid layers near the oxide interface even less hospitable to Bi, and could explain the strong migration of Bi away from that interface for all Bi not trapped above the liquid surface (Figures 3 and S6, Supporting Information).

Looking then to the *g*(*r*) for Au‐Ga and Pt‐Ga (Figures S12b and S12c, Supporting Information), the first and second coordination peaks are consistently shorter than Ga‐Ga by 0.1 and 1.0 Å, respectively. This might imply that although Au and Pt alter the local environment compared to pure gallium, they lack the size‐driven need for extra space that could make migration to the vacuum interface energetically favorable, as described for Bi. The *g*(*r*) for Ag‐Ga and Li‐Ga (Figure S12e,f, Supporting Information), both have first‐neighbor distances nearly identical to Ga‐Ga, with slightly contracted second‐neighbor distances. Using the effective size argument, this may help explain why Ag and Li remain unperturbed in the mid‐surface region: the local environment centered on an Ag or Li dopant changes very little from that of a pure gallium local environment. The migration of all dopants away from the oxide interface could be explained by the relative inflexibility of the gallium network to change–either contract or enlarge – in the surface region nearest that interface.

Although the effective size of each dopant influences interfacial dynamics, it cannot fully explain the observed trends. Sn provides a striking counterexample: despite having bulk coordination peaks of 3.0 and 5.8 Å (Figure S12d, Supporting Information) which closely resemble those of Bi, tin's response to the vacuum interface (Figure S7d, Supporting Information) differs markedly from that of Bi (Figure S7a, Supporting Information). Notably, Bi and Sn are the only dopants whose coordination environments change significantly between the bulk and top‐surface liquid regions (Figures S12a and S12d, Supporting Information), both showing a notable contraction in their first and second nearest‐neighbor distances at the surface. Neither electronegativity nor valency alone can clearly explain this difference, indicating that the dopant response to either interface arises from a complex interplay of multiple factors–in particular, their interaction with gallium's highly structured liquid surface.^[^ ^28^ ^]^ This highlights the importance of comparing dopant interactions with the Ga across distinct liquid environments (top‐surface, mid‐surface, and bulk) to fully understand their differing responses.

**Figure 7:**
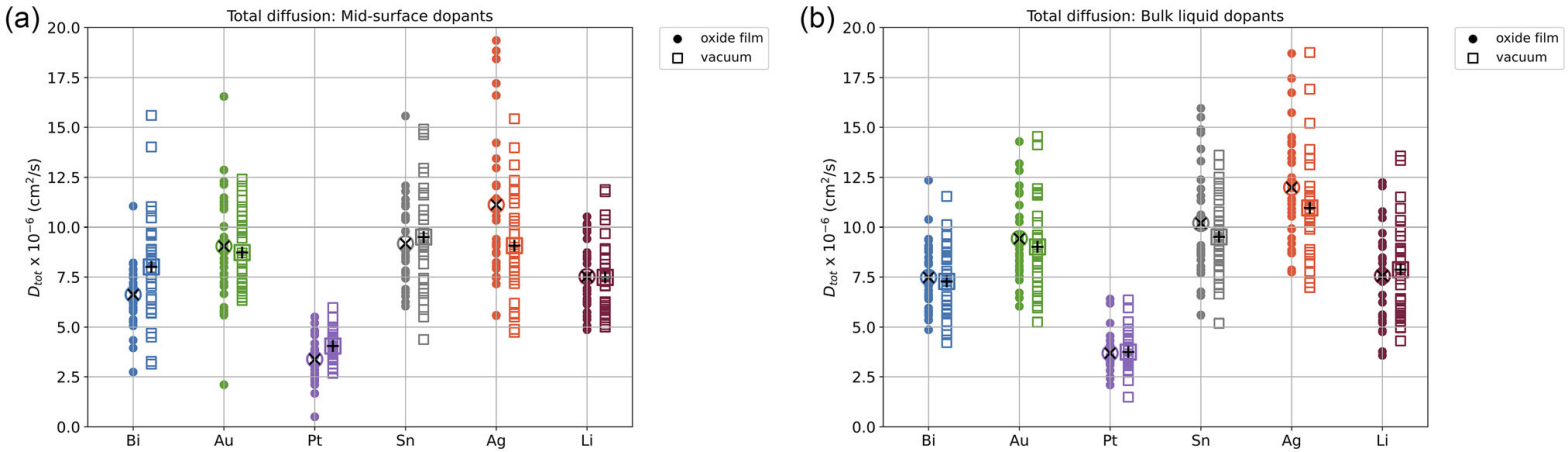
Total diffusion coefficients are shown for low‐dopant ratio (0.055 at%) simulations, where one dopant is initialized in the mid‐surface region. Each data point represents results from 30 simulations per dopant, with separate sets for oxide and vacuum interfaces (30 × 2 × 6 total simulations). In each simulation, two dopants are placed: one in bulk and one in the mid‐surface region, allowing both diffusion behaviors to be extracted from the same run. Diffusion coefficients are given for: a) (filled circles) mid‐surface dopants near an oxide interface; (open squares) mid‐surface dopants near a vacuum interface; b) (filled circles) bulk‐seeded dopants from simulations with a single oxide interface; and open squares) bulk‐seeded dopants from simulations with two vacuum interfaces. Average diffusion coefficients are indicated by (a thick black + in an open square) for mid‐surface vacuum‐seeded dopants and (a thick black × in an open circle) for mid‐surface oxide‐seeded dopants.

As a final analysis, we examine the total diffusion of all dopants to assess whether the oxide film and structured gallium surface influence overall dopant mobility. To eliminate the need to select which of 20 dopants is most representative of a particular liquid region, diffusion rates are calculated from the mid‐surface control simulations. **Figure** **7**a shows the diffusion coefficients for all 30 control simulations for the mid‐surface‐seeded dopants. On average, total diffusion rates for a given dopant are comparable, regardless of whether the dopant is seeded near the vacuum or oxide interface.

Platinum exhibits the lowest overall diffusion, aligning well in magnitude with previous AIMD studies on GaPt slab models.^[^ ^12^ ^]^ Silver has the highest average diffusion along with the greatest variance. Bi, Au, Li, and Sn have similar mobility in the gallium liquid. Figure 7b presents the diffusion coefficients for dopants seeded in the bulk liquid, revealing similar trends with only very small differences in diffusion magnitudes. Since the total diffusion coefficients are comparable across dopants, the previously observed differences in *z*‐mobility become even more significant, highlighting distinct dopant‐specific behaviors in the out‐of‐surface‐plane direction.

## 4 Concluding Remarks

Our simulations reveal distinct dopant behaviors at liquid gallium interfaces, with trends that align well with experimental observations. Bismuth exhibits unique dynamics, showing strong attraction to vacuum interfaces and repulsion from oxide‐coated interfaces. This finding goes a long way toward explaining the experimental findings of Tang et al.^[^ ^4^ ^]^ where bismuth‐rich surface patterns formed in oxidized gallium but disappeared when oxidation was minimal. We propose that oxide wrinkling during cooling causes local detachment from the liquid, creating vacuum interfaces that attract bismuth dopants, while regions with intact oxide repel them. Additionally, the enhanced lateral diffusion of bismuth at vacuum interfaces aligns with the observed enrichment patterns, where bismuth accumulates but spreads across the surface. The results suggest a mechanistic link between oxide wrinkling, dopant trapping, and surface pattern formation, highlighting the critical role of interfacial dynamics in Bi‐doped liquid gallium systems. Understanding these dynamics could help optimize Bi surface patterning in liquid metals for plasmonic or catalytic applications.

In contrast, dopants such as gold, platinum, tin, silver, and lithium display more conventional diffusion behaviors. Platinum's strong Ga–Pt interaction limits its mobility, consistent with experimental findings^[^ ^12^ ^]^ and our previous simulations^[^ ^14^ ^]^ on Pt‐doped gallium liquid systems, while tin's preference for the bulk matches prior experimental observations of surface depletion in Ga–Sn liquids.^[^ ^45^ ^]^ Silver and lithium are repelled by the oxide's structured environment, but remain in the top or mid‐surface layers of gallium at the vacuum interface. Lithium's aversion to the oxide interface, coupled with its significantly increased mobility at the vacuum interface, could have significant implications for the design of low‐temperature liquid metal batteries, potentially influencing their viability and technological development. Together, these findings highlight how interfacial behavior varies across dopants and suggest strategies for tailoring liquid metal systems depending on whether surface enrichment or bulk retention is desired.

Although bismuth and tin share similar covalent radii, their markedly different interfacial behaviors underscore that atomic size alone does not govern dopant segregation trends. This highlights the importance of chemical identity and interface structure in shaping dopant dynamics. Future work will expand on this by exploring additional elements with comparable radii but distinct electronic characteristics, such as Cu or Zn, to further probe the interplay between size, chemistry, and interfacial preference. We also plan to develop MLFF models at higher dopant concentrations to explore the effects of increased dopant levels on dynamics and interfacial behavior.

Together, these findings demonstrate the rich interfacial physics that emerges from dopant–liquid–interface interactions, and underscore the value of MLFF simulations in accessing atomistic mechanisms that are difficult to isolate experimentally. The observed behaviors are not easily explained by atomic size or charge alone, and arise from a complex interplay of factors—including valency, chemical affinity, and the structuring of the interface itself. In the course of our analysis, we explored several simple descriptors, including valency, mass‐to‐charge ratio, and effective radii (estimated from the peaks in the radial distribution function, RDF), but found that no single factor—or even simple pair of factors—consistently explained the trends across all six dopants. Each hypothesis had at least one clear counterexample within the six tested dopants. These results highlight the complexity of the electronic and structural factors that govern dopant distribution within the Ga network, and underscores the need for interatomic potentials generated from high‐level ab initio calculations. Future work will include additional dopants and further methods to couple electronic structure and MLFF simulations. Our results make it clear, however, that future design of liquid metal systems for catalysis, electronics, or energy applications must consider not only bulk composition, but also the nature of interfacial environments and their influence on dopant dynamics.

## 5 Experimental Section

All simulations employ an MLFF trained using VASP 6.4.2^[^ ^46^, ^47^, ^48^, ^49^ ^]^ by the on‐the‐fly machine learning algorithm.^[^ ^37^, ^38^, ^39^ ^]^ The density functional theory training steps employ the projector augmented wave method,^[^ ^50^, ^51^ ^]^ the smallest allowed spacing between *k*‐points of 0.25 Å^−1^ (Γ‐centered), and the PBE for solids (PBEsol) exchange‐correlation functional,^[^ ^52^ ^]^ which has been shown to accurately capture the properties of liquid gallium across a wide‐range of temperatures.^[^ ^33^ ^]^ A separate MLFF is trained for each of the six dopants: Ag, Au, Bi, Li, Pt, and Sn. Each MLFF is used to perform MD simulations of doped liquid gallium systems with 3608 liquid atoms (3586 liquid gallium, 20 dopants yielding a 0.55 at % dopant ratio). The unit cells are periodically replicated in two dimensions, with vacuum added to the third (*z*) dimension. A six‐layer film of *α*‐Ga_2_O_3_ (768 Ga, 1152 O) is added to one of the liquid interfaces, while the other interface remains vacuum. The *α*‐Ga_2_O_3_ phase is selected due to its reported stability as a thin film near our simulated temperature,^[^ ^53^, ^54^, ^55^ ^]^ as well as its well‐defined structure, which facilitates controlled comparison of dopant–interface interactions. While more disordered oxide structures may influence interfacial behavior, they also introduce additional complexity by convoluting dopant statistics with those of the oxide configurations, which is a challenge we leave for future work. The simulations are thermostatted^[^ ^56^, ^57^, ^58^, ^59^ ^]^ to 450 K and are run for 200 ps with a time step of 1 fs.

The initial depth (${\bar{z}}_{i}(t=0)$), defined as the distance from the liquid surface closest to the oxide interface, for each of the 20 dopants is distributed approximately evenly between the two interfaces, as illustrated in Figure 1. The placement is systematic, with the first dopant closest to the oxide, the second slightly farther away, etc. We complete 30 MD simulations for each of the 6 dopant types (180 simulations). In all simulations, the initial dopant depth remains approximately unchanged but their initial lateral positions are varied. This prevents observed dopant behavior from being overly influenced by the random choice of initial position. The systematic initialization of depth allows for the calculation of an average depth per dopant atom (*i*) at each MD time step, ${\bar{z}}_{i}(t)$ (more detail in Section S2).

Five additional sets of *control* MLFF simulations (30 runs each set) are also performed. The first set of control simulations, labeled *double‐vacuum*, uses the same setup as the main simulations (3586 liquid gallium atoms, 20 dopant atoms, 450 K, 1 fs time step), but removes the oxide film. These simulations confirm that observed trends at the vacuum interface are not influenced by the presence of the oxide at the opposite interface. The four remaining control simulations have an order of magnitude lower dopant ratio (0.055 at %), with two dopants in 3606 liquid gallium atoms. These simulations have one dopant placed midway between the two interfaces, while the position of the second dopant varies: 1) the second dopant is seeded in the top‐surface layer nearest the gallium oxide film; 2) the second dopant is seeded in the top‐surface layer near one of two vacuum interfaces; 3) where the second dopant is seeded in the mid‐surface layer region nearest the oxide film; or 4) the second dopant is seeded in the mid‐surface layer region near one of two vacuum interfaces. Results from each of the control simulations consistently support the dynamic trends observed for the main (20‐dopant, oxide‐interface) simulations. Additional details of all simulation methods as well as training and benchmarking of each MLFF can be found in Sections S1 and S2, respectively.

## Conflict of Interest

The authors declare no conflict of interest.

## Author Contributions

**Krista G. Steenbergen**: conceptualization (equal); data curation (lead); formal analysis (lead); funding acquisition (supporting); investigation (lead); methodology (lead); validation (equal); visualization (lead); writing—original draft (lead); writing—review and editing (lead). **Stephanie Lambie**: conceptualization (equal); validation (equal); writing—original draft (supporting); writing—review and editing (supporting). **Charlie Ruffman**: conceptualization (equal); validation (equal); writing—original draft (supporting); writing—review and editing (supporting). **Nicola Gaston**: conceptualization (equal); funding acquisition (equal); project administration (lead); validation (equal); writing—original draft (supporting); writing—review and editing (supporting).

## Supporting information

Supplementary Material

## Data Availability

The data that support the findings of this study are available from the corresponding author upon reasonable request.
