# Supplementary material for: Atomic‐Scale Dynamics at the Interface of Doped Liquid Gallium: Contrasting Effects of Gallium Oxide and Vacuum
Source: Small Sci. 2025 May 15;5(6):2500153. doi: 10.1002/smsc.202500153 (PMC12168597; doi:10.1002/smsc.202500153)
Supplement: Supplementary file 1 — Supplementary Material [file SMSC-5-2500153-s001.pdf]

Supporting information

S1 All simulation details

S1.1 General settings

We utilise VASP's on-the-fly force field training for all MLFF generation.[37–39] Using VASP 6.2.4,[46–49] all pure density functional theory (DFT) calculations employ the projector augmented wave (PAW) method,[50,51] with the smallest allowed spacing between k-points of 0.25 Å^{-1} (Γ-centred). We use the PBE for solids (PBEsol) exchange correlation functional,[52] which has been shown to accurately capture the properties of liquid gallium across a wide-range of temperatures.[33]

For those simulations that include a gallium oxide film, the experimentally determined phase stability was considered. Ga_2O_3 stability depends on both the dimensionality (thin film vs. bulk) as well as the thermodynamic conditions. Experiment has demonstrated that in bulk form, β-Ga_2O_3 is stable at standard temperature and pressure. However, as a thin film at $p = 0$, the stable temperature ranges are: 300-500 K for amorphous Ga_2O_3; 500-700 K for α-Ga_2O_3; and above 800 K for for β-Ga_2O_3.[53–55] Given that our simulations capture the oxide film at 450 K, this sits close to the boundary between the experimentally measured amorphous and α-Ga_2O_3 structures. In order to avoid the complexity of an ill-defined amorphous structure, we select the α-Ga_2O_3 phase for our simulations. This is likely a reasonable assumption of structure, especially given that the temperature range of stable phases in computational simulations is often slightly offset from experimental values.

Since gallium oxide remains solid at 450 K which prevents the unit cell from collapsing, all MD simulations with an oxide film are performed in the NpT ensemble ($p = 0$) allowing the oxide to respond to the liquid. The unit cell is constrained such that the a and b dimensions vary while the c dimension (vacuum direction) remains fixed. These simulations use the Langevin thermostat[56,57] and Parinello-Rahman barostat.[58,59] In contrast, simulations without an oxide film consist solely of liquid, making these systems highly fluxional. Without a solid framework, a flexible cell would lead to excessive deformation, so the liquid-only simulations are performed in the NVT ensemble with a rigid unit cell and a Langevin thermostat.[57]

S1.2 MLFF training

We systematically develop an accurate MLFF for both pure and doped liquid gallium interfaced with an oxide film. A separate MLFF is trained for each of the six dopants, Ag, Au, Bi, Li, Pt, and Sn, with the training proceeding in six steps. At each training step, the local reference configurations from the previous step are used as input for continued training. Training included six different structural configurations: (1) a bulk crystalline α-Ga_2O_3 structure (Fig. S1a); (2) a 6 layer thin film of α-Ga_2O_3 interfaced with a gallium liquid (Fig. S1b); (3) the α-Ga_2O_3 thin film interfaced with a gallium liquid, including one dopant atom seeded into the top layer of the gallium liquid (just beneath the oxide) and the other at the liquid-vacuum interface (Fig. S1c); (4) the α-Ga_2O_3 thin film and gallium liquid, with one dopant in the sub-surface liquid layer from both the oxide and vacuum interfaces (Fig. S1e); (5) the α-Ga_2O_3 thin film with four dopant atoms seeded near the z-centre of the gallium liquid, and one dopant seeded at the liquid-vacuum interface (Fig. S1d). In training step (6), all local reference configurations, energies, forces and stress tensors stored from these five steps are used to re-select basis functions using higher-accuracy settings, and the force field is then refit for "fast" evaluation.

For all MLFF training steps, each configuration is annealed from 450 K to 600 K. Training steps 1 and 2 are common to all MLFFs; however, steps 3-6 are unique for each of the six dopant types. Each dopant-

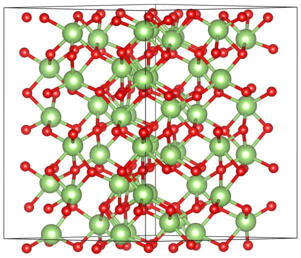

(a) Training step 1: Bulk GaOx

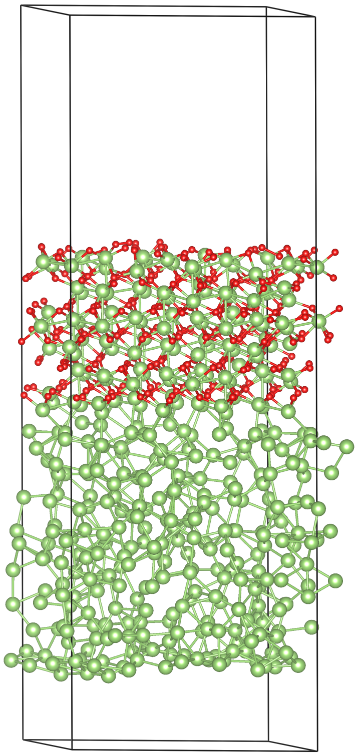

(b) Training step 2: Oxide + liquid

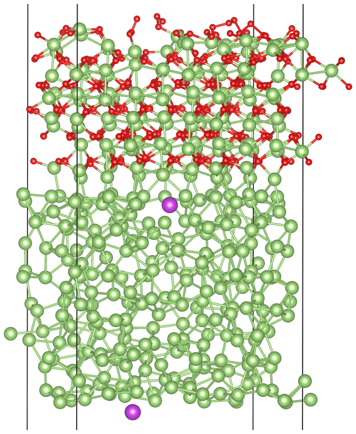

(c) Training step 3: Oxide + liquid + top-surface-oxide & top-surface-vacuum dopants

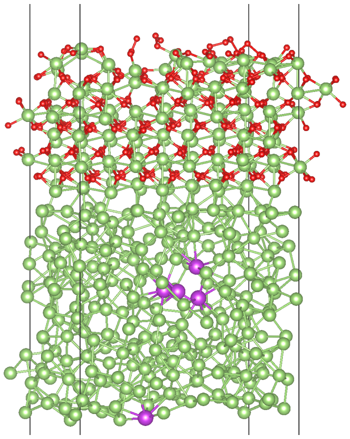

(d) Training step 4: Oxide + liquid + top-surface-vacuum & bulk-liquid dopants

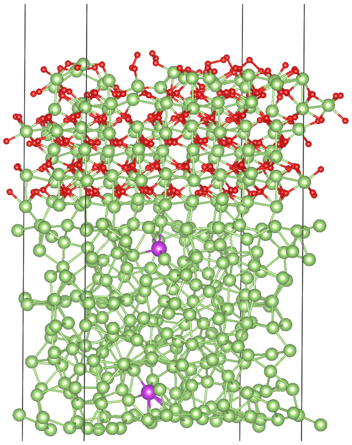

(e) Training step 5: Oxide+ liquid + mid-surface-oxide & mid-surface-vacuum dopants

Figure S1: Snapshots of the configurations used for the first 5 MLFF training steps. The 6th step is a re-fitting of the force field based on the accumulated local reference configurations, and therefore includes contributions from each of these 5 configurations.

specific MLFF development follows the same training methodology, but dopant types are never mixed. Further details of each MLFF training step are given here.

Step 1, Crystalline Ga_2O_3: The α-Ga_2O_3 training supercell has 48 gallium and 72 oxygen atoms, with dimensions $a = b$=10.1 Å, c=13.6 Å and $\alpha = \beta = 90°$ and $\gamma = 120°$. The calculation is periodic in all three dimensions, with the geometry illustrated in Fig. S1a. The supercell was optimised, annealed to 450 K, and equilibrated at 450 K using pure DFT for 1 ps. The thermalised post-equilibration geom-

etry is used to initialise an MLFF on-the-fly training simulation of 500 time steps (Δt=2 fs) in the NpT ensemble, while annealing from $T = 450$ K to $T = 600$ K.

Step 2, Thin film Ga_2O_3 + Ga liquid: Here, a 6-layer thin film of α-Ga_2O_3 is interfaced with a gallium liquid. The simulation supercell has dimensions of a=20.2 Å, b=10.1 Å, c=60.6 Å and $\alpha = \beta = 90°$ and $\gamma = 120°$, and includes 96 gallium and 144 oxygen atoms in the oxide, with 184 liquid gallium atoms. The calculation is periodic in the x and y directions, but has two vacuum-interfaces in the z-dimension: one oxide-vacuum interface and one gallium liquid-vacuum interface (Fig. S1b). The supercell is equilibrated at $T = 450$ K in the NpT ensemble using pure DFT for 0.5 ps. The equilibrated configuration is then used to initialise an on-the-fly MLFF training run, which included 1000 time steps (Δt=1.5 fs) in the NVT ensemble, annealing from $T = 450$ K to $T = 600$ K. In order to better capture the complexity of the gallium liquid state, the maximum number of local reference configurations is increased to 3000. A total of 133 configurations were saved from this training step.

Dopant-specific FF: Here, the MLFF training diverges for each of six dopant types: Ag, Au, Bi, Li, Pt, and Sn. However, each dopant-specific MLFF is initialised from the same set of local reference configurations (obtained from steps 1 and 2), and follows the same training methodology, with the only difference being the type of dopant atom used in the simulation. In the descriptions below, we use the label X to refer to one of the six dopant elements, noting that dopant types are never mixed during training.

Step 3, Oxide + Ga liquid and top-surface dopants: From the supercell of Step 2, two liquid gallium atoms are exchanged for dopant atoms, creating a 182 atom gallium liquid with 2 X dopants. One dopant is seeded at the top-surface layer nearest the oxide and the other in the top-surface near the vacuum interface (Fig. S1c). The new configuration is equilibrated at $T = 450$ K in the NVT ensemble using pure DFT for 0.5 ps. As testing showed that the oxide film changed very little, the z-coordinate of the top 3 layers of the oxide film is held fixed, allowing the bottom layers to change (if needed) in response to the dopant but permitting an increase in the simulation time step for greater sampling of the liquid-dopant configuration space. (We note, however, that in all production MLFF simulations reported in the manuscript, the oxide layer is not frozen, allowing for structural accommodation and interfacial interactions during dopant dynamics.) Following pure DFT, on-the-fly MLFF training is performed for 500 time steps (Δt=6 fs) in the NVT ensemble, annealing from $T = 450$ K to $T = 600$ K. The maximum number of local reference configurations remained at 3000.

Step 4, Oxide + Ga liquid + bulk and top-surface dopants: From the supercell of Step 2, five dopant atoms are substituted for gallium liquid atoms, creating a 179 atom gallium liquid with 5 X dopants. In an attempt to capture aggregate behaviour of the dopant atoms, four X atoms are seeded near the z-centre of the gallium liquid. One dopant is again seeded at the top-surface layer near the vacuum interface (Fig. S1d). All other settings and steps remain the same as Step 3.

Step 5, Oxide + Ga liquid + mid-surface layer dopants: For certain dopants, the dopant atom seeded at an interface did not migrate to the mid-surface layers for the duration of all MD training steps. As our previous work indicated that there are 3 distinct surface liquid layers that have a distinct geometrical ordering from the bulk liquid, we wanted to ensure our training covered mid-surface layer interactions as well. Therefore, this fifth training step again modifies the supercell from step 2, seeding one dopant in the 2nd liquid layer from both the oxide and vacuum interfaces (Fig. S1e). For this step, the maximum number of local reference configurations is increased to 3300.

Step 6, Re-selection and refit for increased accuracy: Finally, all configurations, energies, forces and stress tensors stored from the previous 5 training steps are used to re-select local reference configurations using higher-accuracy settings. Here, the cutoff radius for the angular descriptor is increased to 5.4 Å (default 5.0 Å), the maximum angular momentum quantum number is increased to 4 (default 3), and the maximum number of local reference configurations is increased significantly to 4400. The higher-accuracy settings were not employed for steps 1-5 due to calculation (memory) limitations. Following the

re-selection, the force field is then refit for "fast" evaluation.

Table S1: Total number of local reference configurations (LRCs) for each atomic species in each doped system, as extracted from the the Step 6 re-selection ML_LOGFILE.

System	Ga LRCs	O LRCs	Dopant LRCs
GaAg	4216	3636	401
GaAu	4169	3588	399
GaBi	4422	4130	414
GaLi	3851	3572	355
GaPt	4457	3683	415
GaSn	4419	3680	418

Table S2: Mean energy, force, and stress RMSEs for each doped system, computed from the predictions with respect to *ab initio* results for the configuration immediately preceding each *ab initio* reference calculation.

System	Energy RMSE (10^{-4} eV)	Force RMSE (10^{-2} eV/Å)	Stress RMSE (10^{-1} kB)
GaAg	5.72	6.92	4.12
GaAu	5.39	6.94	4.11
GaBi	5.60	6.59	4.69
GaLi	5.00	6.70	4.18
GaPt	6.92	6.94	3.97
GaSn	5.48	6.73	3.86

S1.3 MLFF simulations

MLFF training proceeded in six systematic steps, with a separate MLFF trained for each of the six dopants: Ag, Au, Bi, Li, Pt, and Sn. All details of the MLFF training are included in the supporting information (Section S1.2). The finalised MLFFs are used to complete large MD simulations of doped liquid gallium systems. These simulations have a unit cell of approximately a = 40.5 Å and b = 40.35 Å for the periodically replicated dimensions, with $\alpha = \beta = 90°$, $\gamma = 120°$ for the lattice angles. A vacuum is added in third unit cell dimension, giving two liquid interfaces, which are oriented perpendicular to the z-axis of the simulation unit cell. The simulations are thermostatted to 450 K and use a time step of 1 fs. Each simulation was run for 201 ps simulation time (1 ps equilibration, 200 ps production). Geometric data was recorded every 100 fs, yielding 2000 geometric snapshots.

All MLFF simulations include a total of 3608 liquid atoms. The primary simulations consist of 3586 liquid gallium atoms with 20 dopant atoms (0.55 at. % dopant ratio). Each simulation contains *only one dopant type*, with separate simulations run for Au, Ag, Bi, Li, Pt, and Sn. A six-layer film of α-Ga_2O_3 (768 Ga, 1152 O) is added to one of the liquid interfaces, while the other interface remains a vacuum. The initial z position of each of the 20 dopants is distributed approximately evenly between the two interfaces, as illustrated in Fig. 1. The first dopant is placed closest to the oxide or top-vacuum interface, the second slightly farther away, and so on, with the 20th dopant positioned near the opposite interface.

We complete 30 different MD simulations for each of the 6 dopant types (180 simulations). Each simulation keeps the initial depth of the dopant approximately unchanged, but varies the lateral position (x and y coordinates) of the dopants. This prevents observed dopant behaviour from being overly influenced by the random choice of initial position. The systematic placement (first dopant closest to the oxide, etc.) allows meaningful correlation of dopant behaviour and dynamics across all 30 simulations.

S1.3.1 Control simulations

We also complete five additional sets of *control* MLFF simulations. Each control set consists of 30 different MD simulations, where the initial depth of each dopant is kept approximately constant for all simulations, but the initial lateral positions are varied. The first set of control simulations uses the same setup as the main simulations (3586 liquid gallium atoms, 20 dopant atoms, 450 K, 1 fs time step), but removes the oxide film. These *double-vacuum* simulations serve as an essential reference to confirm that dopant trends observed at the vacuum interface in the main simulations are not influenced by the presence of the oxide at the opposite interface.

In order to verify that the dynamics observed with 20 dopants remain consistent at lower dopant concentrations (where dopants are widely separated), we conducted four additional sets of *control* MLFF simulations. These simulations utilise the same simulation parameters (450 K, 1 fs time step) and also contain 3608 liquid atoms, but have only two dopants in 3606 liquid gallium atoms, resulting in a 0.055 at. % dopant ratio (an order of magnitude lower than in the main simulations). In all control simulations, one dopant is placed at the midpoint between the two interfaces, while the position of the second dopant varies across four distinct sets of control simulations: (1) the second dopant is seeded in the top-surface layer nearest the gallium oxide film; (2) the second dopant is seeded in the top-surface layer near one of the two vacuum interfaces; (3) the second dopant is seeded in the mid-surface layer region nearest the oxide film; or (4) the second dopant is seeded in the mid-surface layer region near one of the two vacuum interfaces.

S2 Analysis and benchmarking

The results section presents two primary analyses: the directional diffusion coefficients and the average depth of dopant atoms. All diffusion coefficients are calculated from the initial slope of the mean squared displacement (MSD) curves using the Einstein relation. Directional diffusion is the diffusion measured in each of the Cartesian directions. Supporting Information Section S2.1 gives the equations and additional details of this calculation.

The *depth* of a dopant, z, represents its perpendicular distance from the upper boundary of the gallium liquid (as shown in Fig. 1). This boundary is defined as the average z-coordinate of the 20 liquid gallium atoms closest to the oxide film. The average depth, $\bar{z}(t)$, is the depth of a given dopant averaged across multiple MD simulations at each MD time step, t. Let i index dopant atoms ($i = 1, 2, ..., 20$) and j index MD simulations ($j = 1, 2, ..., 30$). At each time step, t, the average depth of dopant i is given by:

$$\bar{z}_i(t) = \frac{\sum_{j=1}^{30} z_{i,j}(t)}{30} \quad . \tag{1}$$

Since each dopant i is initially seeded at approximately the same depth across all 30 simulations, the averaging of its dynamic behaviour reflects how the dopant responds to the seeded liquid region.

Benchmarking of pure liquid gallium has been previously completed, showing excellent agreement between full DFT and MLFF simulations.[28] Here, we focus on dopant-specific benchmarking. To assess each newly developed MLFF, 5 ps AIMD simulations are conducted on a smaller supercell containing 274 liquid Ga atoms (without oxide), with one dopant placed at both the top surface and one in the bulk region of the liquid. Radial distribution functions for dopant-Ga distances are computed to ensure that the structural environment around a dopant aligns between the MLFF and AIMD simulations. For GaBi, the AIMD simulation is extended to 68.4 ps to obtain a meaningful measure of mean squared displacement (MSD). While these AIMD supercells are smaller than their MLFF counterparts, they are still computationally demanding due to the high cost of DFT. As a result, performing similarly long AIMD sim-

ulations for all six dopants was prohibitive. Additional benchmarking details and results are provided in Supporting Information, Section S3.

S2.1 MSD and diffusion calculations

The mean squared displacement (MSD) is calculated using multiple time origins to improve statistical accuracy. Specifically, the calculation included a total of $\Delta T/2$ time origins, where ΔT is the total length of the molecular dynamics (MD) simulation. Time origins were selected from the first half of the MD simulation, ensuring that each origin had sufficient subsequent trajectory to calculate the MSD at the desired time intervals. This approach provides a robust representation of the dynamic behaviour over the simulation time while avoiding artifacts introduced by the trajectory's finite length.

In general, the MSD is calculated over multiple time-origins per individual dopant atom, i, as:

$$\mathrm{MSD}_i(t) = \frac{1}{N_t} \sum_{t_0=0}^{N_t-1} \left|\mathbf{r}_i(t_0+t) - \mathbf{r}_i(t_0)\right|^2 \tag{2}$$

where:

- t is the elapsed time after the time origin t_0.
- $\mathrm{MSD}_i(t)$ is the MSD of dopant i at elapsed time t.
- t_0 represents the different time origins used for the calculation.
- N_t is the total number of time origins ($\Delta T/2$).
- $\mathbf{r}_i(t)$ is the position of particle i at time t.

The total mean squared displacement (MSD) for a single particle can be expressed as the sum of its directional components (x, y, and z). Recognising that the term to the right of the sum can be broken into each Cartesian component as:

$$\left|\mathbf{r}_i(t_0+t) - \mathbf{r}_i(t_0)\right|^2 = \left(r_{i,x}(t_0+t) - r_{i,x}(t_0)\right)^2 + \left(r_{i,y}(t_0+t) - r_{i,y}(t_0)\right)^2 + \left(r_{i,z}(t_0+t) - r_{i,z}(t_0)\right)^2 \quad , \tag{3}$$

then the total MSD can be explicitly written as:

$$\mathrm{MSD}_i(t) = \frac{1}{N_t} \sum_{t_0=0}^{N_t-1} \left[\left(r_{i,x}(t_0+t) - r_{i,x}(t_0)\right)^2 + \left(r_{i,y}(t_0+t) - r_{i,y}(t_0)\right)^2 + \left(r_{i,z}(t_0+t) - r_{i,z}(t_0)\right)^2 \right]. \tag{4}$$

We can then define the individual directional MSDs:

$$\mathrm{MSD}_{i,x}(t) = \frac{1}{N_t} \sum_{t_0=0}^{N_t-1} \left(r_{i,x}(t_0+t) - r_{i,x}(t_0)\right)^2 , \tag{5}$$

$$\mathrm{MSD}_{i,y}(t) = \frac{1}{N_t} \sum_{t_0=0}^{N_t-1} \left(r_{i,y}(t_0+t) - r_{i,y}(t_0)\right)^2 , \tag{6}$$

$$\mathrm{MSD}_{i,z}(t) = \frac{1}{N_t} \sum_{t_0=0}^{N_t-1} \left(r_{i,z}(t_0+t) - r_{i,z}(t_0)\right)^2 . \tag{7}$$

Finally, the total MSD is the sum of the three components:

$$\mathrm{MSD}_i(t) = \mathrm{MSD}_{i,x}(t) + \mathrm{MSD}_{i,y}(t) + \mathrm{MSD}_{i,z}(t). \tag{8}$$

The diffusion coefficient (D) is calculated using the Einstein relation, which relates the mean squared displacement (MSD) to the linear regime of particle motion. For the total diffusion coefficient, D, the expression is:

$$D = \lim_{t\to\infty} \frac{\mathrm{MSD}_i(t)}{6t}, \tag{9}$$

where:

- $\mathrm{MSD}_i(t)$ is the total mean squared displacement of dopant i,
- t is the time,
- The factor of 6 assumes the three translational degrees of freedom.

In this work, the linear regime of the MSD was assumed to hold for the first 10 ps of the simulation, and D was determined by taking the slope of $\mathrm{MSD}_i(t)$ versus time in this regime. Using total MSD expressed in terms of its directional components as given in Eq. 8, we can then rewrite the total diffusion coefficient as:

$$D = \lim_{t\to\infty} \frac{\mathrm{MSD}_{i,x}(t) + \mathrm{MSD}_{i,y}(t) + \mathrm{MSD}_{i,z}(t)}{6t}. \tag{10}$$

Since each directional MSD contributes equally in an isotropic system (with 2 translational degrees of freedom per direction), the diffusion coefficient in each direction is:

$$D_x = \lim_{t\to\infty} \frac{\mathrm{MSD}_{i,x}(t)}{2t}, \quad D_y = \lim_{t\to\infty} \frac{\mathrm{MSD}_{i,y}(t)}{2t}, \quad D_z = \lim_{t\to\infty} \frac{\mathrm{MSD}_{i,z}(t)}{2t} \quad . \tag{11}$$

By calculating the slope of $\mathrm{MSD}_{i,x}(t)$, $\mathrm{MSD}_{i,y}(t)$, and $\mathrm{MSD}_{i,z}(t)$ in the linear regime (the first 10 ps), the directional diffusion coefficients (D_x, D_y, D_z) can be determined as 1/2 of the MSD(t) slope. The total diffusion coefficient is given by the average of the directional components:

$$D = \frac{D_x + D_y + D_z}{3}. \tag{12}$$

Sometimes, the diffusion coefficients (directional and total) are given as averages across multiple simulations. In this case, taking i as the atom index and j as the index representing simulation number,

$$\overline{D}_i = \frac{\sum_{j=1}^{30} D_{i,j}(t)}{30} \quad . \tag{13}$$

Since the atoms are seeded into each simulation systematically according to their depth and liquid regions, each i represents an atom seeded into the same liquid layer in all 30 simulations. The average directional diffusion coefficients, $\overline{D}_x$, $\overline{D}_y$ and $\overline{D}_z$, are calculated by the same equation, substituting one of the directional components for D on both sides of the equation.

S3 Benchmarking MLFF against full DFT

The full DFT GaBi simulations also employ the PBEsol exchange correlation functional. These simulations are completed for the doped gallium liquid system (no oxide), with 299 gallium liquid atoms and 2 bismuth. One bismuth was seeded in the top-surface layer and one bismuth was seeded in the middle of the liquid – the configuration correlates to the top-seeded control MLFF simulations, although having a much smaller configurational size. The supercell has h-matrix vectors of: $\vec{a} = (16.4, 0.0, 0.0)$, $\vec{b} = (-8.1, 14.2, 0.0)$, $\vec{c} = (0.0, 0.0, 60.0)$. These simulations are run with a time step of 6 fs for 11,400 DFT-MD time steps (68.4 ps).

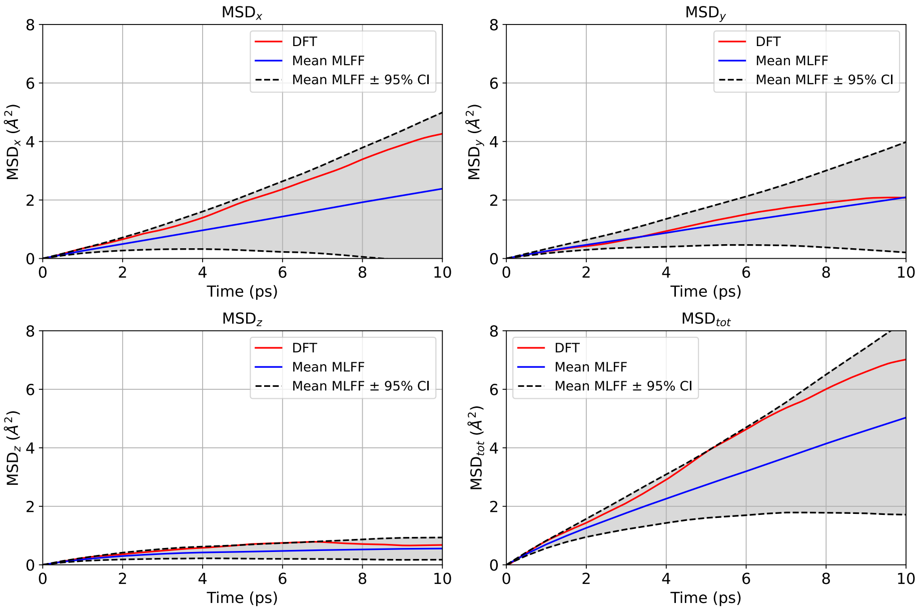

(a) Top-seeded Bi

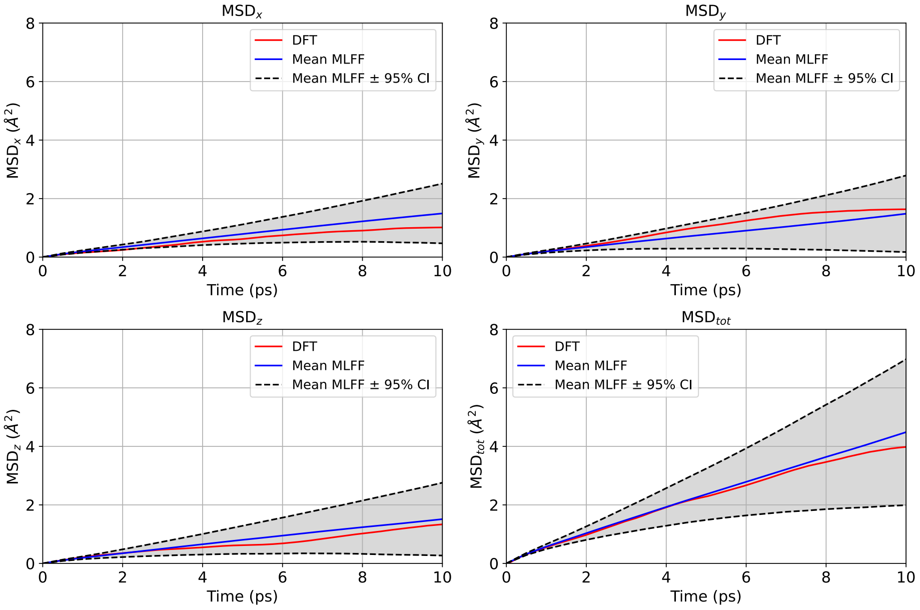

(b) Bulk-seeded Bi

Figure S2: Directional and total MSD curves for the full DFT (red) simulation benchmarked against the average MSD (blue) of the Bi**TsB** MLFF simulations. We also include the 95% confidence interval window (shaded grey). We give the directional MSD results for (a) the top-seeded Bi and (b) the bulk-seeded Bi.

Examining the MSD measurements from full DFT compared to the MLFF results in Fig. S2, the MLFF-

predicted MSD falls within the 95% confidence interval across all directions for both top- and bulk-seeded atoms. This confidence interval is defined as the mean ± two standard deviations, calculated from the directional MSDs of 50 MLFF control simulations. We note that the general trends are nearly identical between the two sets of simulations, where the z-mobility of the top-seeded atom is exceptionally low (Fig. S2a, MSD_z). Due to the comparably small simulation cell for the full DFT simulations, the minor differences are attributed to finite size effects in the full DFT simulations.

For local environment comparisons, 5 additional full DFT simulations are completed. These simulations have 299 gallium liquid atoms and 2 X dopant (X=Pt, Au, Sn, Li, Ag), with the same supercell dimensions given above for the Bi full DFT simulation. These simulations are run with a 6 fs time step, but are far shorter with only 6 ps total simulation time. The simulation set-up once again correlates to the top-seeded control MLFF simulations.

Figs. S3 and S4 show the distribution of X–Ga bond lengths within the local environment of top-seeded and bulk-seeded dopants (plotted separately), comparing the results from the DFT and MLFF top-seeded control simulations. The distributions were obtained by calculating the bond lengths between each dopant and surrounding Ga liquid atoms. Since each $g(r)$ is only measuring the coordination environment around a single atom, which is then averaged over 1000 time time steps, the $g(r)$ for an individual simulation is quite noisy and varied considerably between the 50 MLFF control simulations. We therefore present a window around each MLFF $g(r)$, representing one standard deviation, calculated from the $g(r)$ of 50 MLFF control simulations

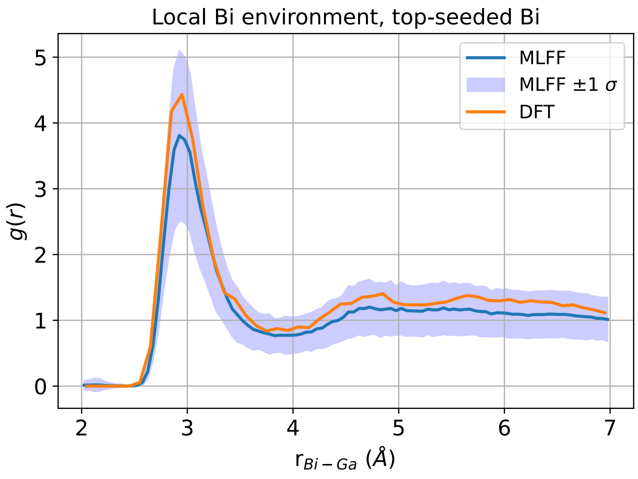

(a) Top-seeded Bi

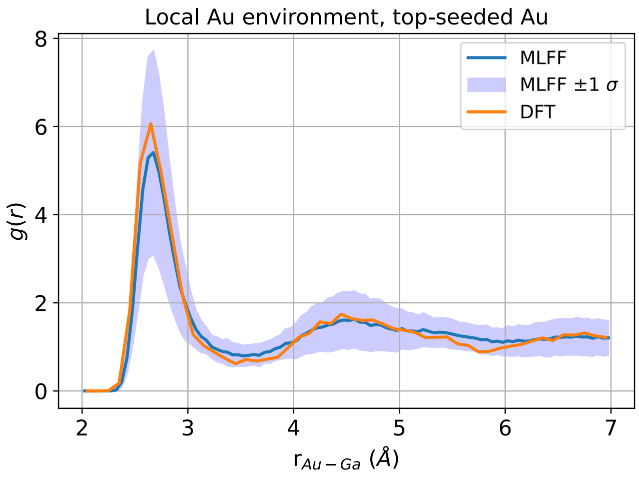

(b) Top-seeded Au

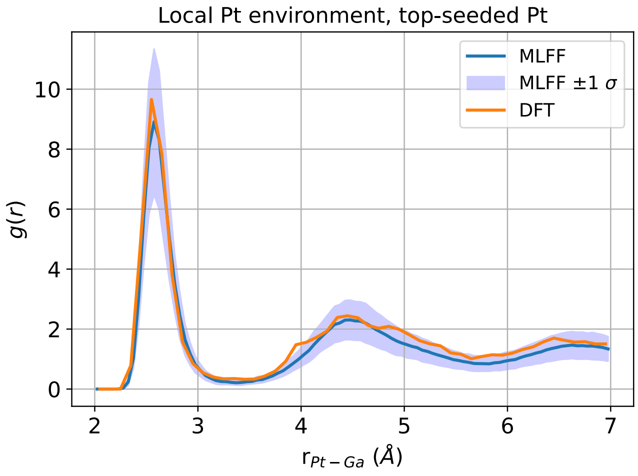

(c) Top-seeded Pt

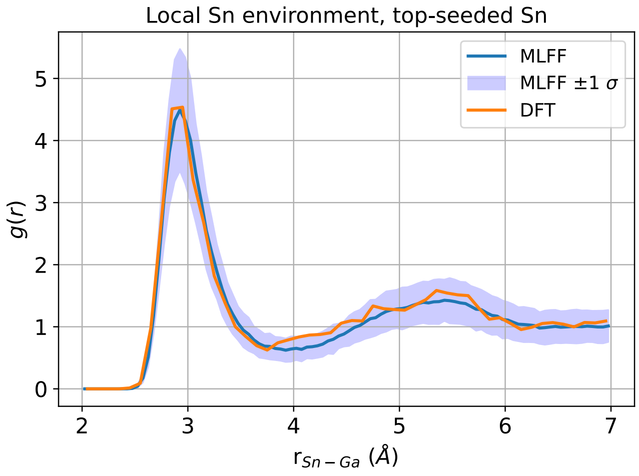

(d) Top-seeded Sn

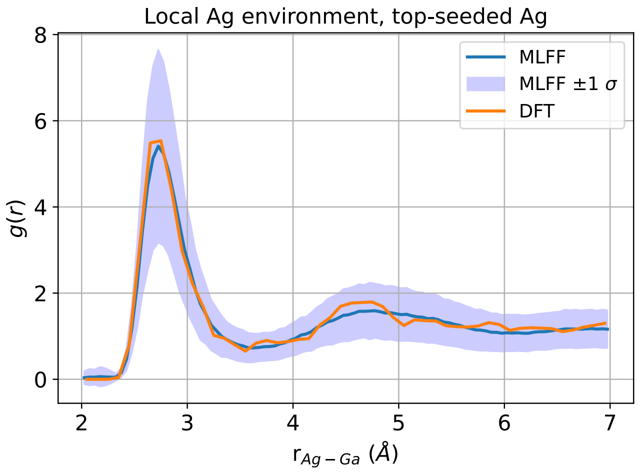

(e) Top-seeded Ag

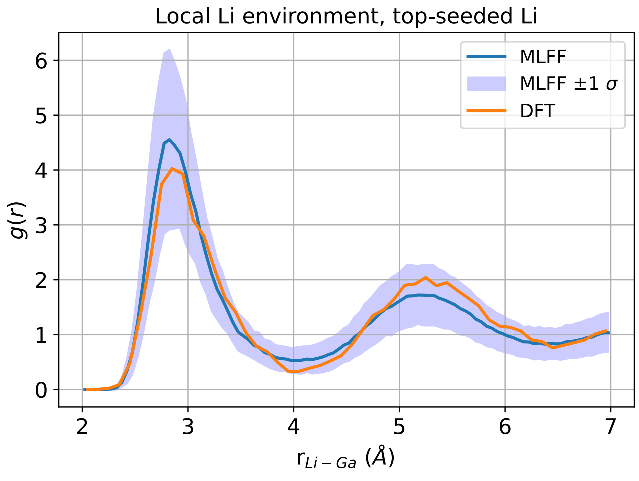

(f) Top-seeded Li

Figure S3: Pair distribution functions of X–Ga for all top-seeded dopants from DFT (red lines) and MLFF (blue lines) simulations with double-vacuum. Note that the shell-volume normalization for $g(r)$ is based on half the full spherical shell volume, reflecting the fact that atoms at the vacuum interface experience approximately half of a coordination shell due to the absence of neighboring atoms in the vacuum.

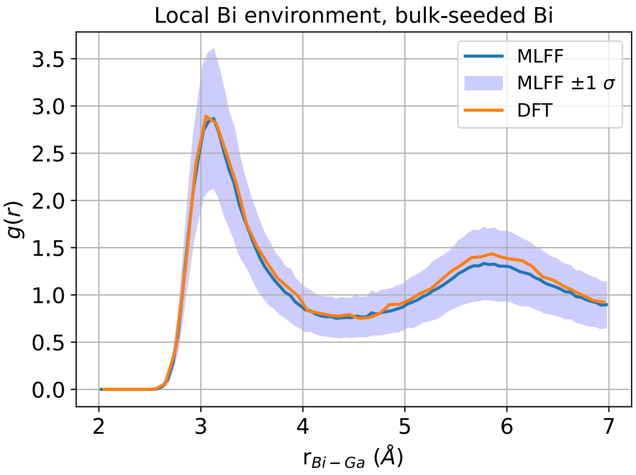

(a) Bulk-seeded Bi

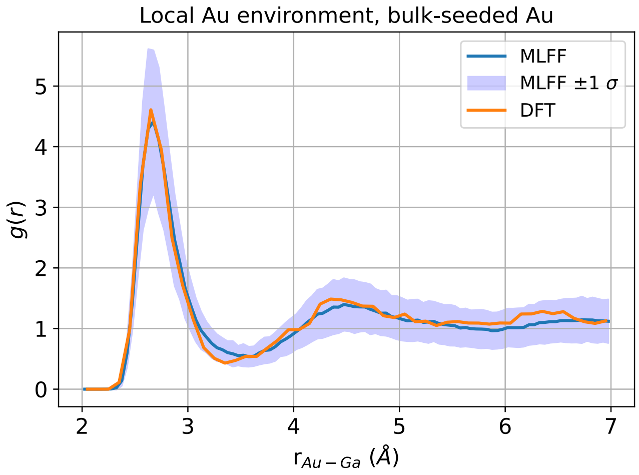

(b) Bulk-seeded Au

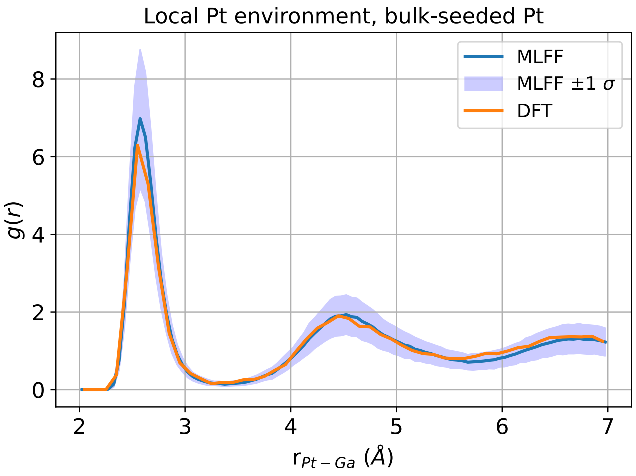

(c) Bulk-seeded Pt

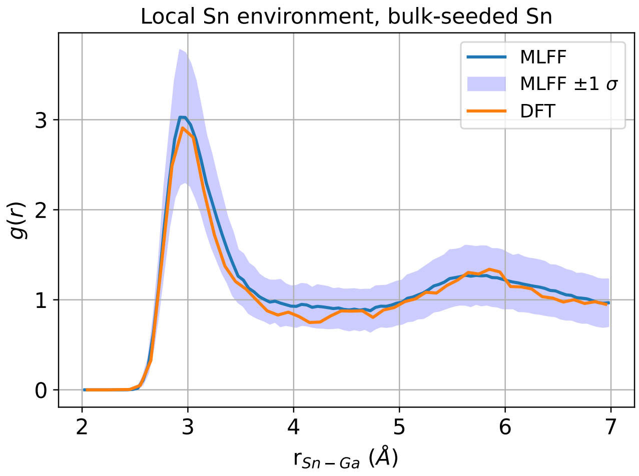

(d) Bulk-seeded Sn

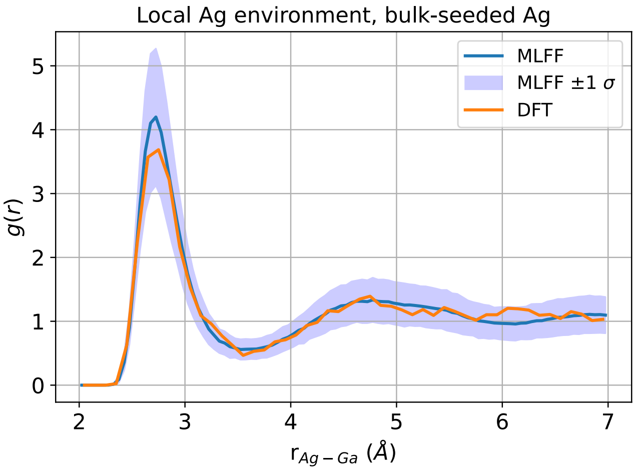

(e) Bulk-seeded Ag

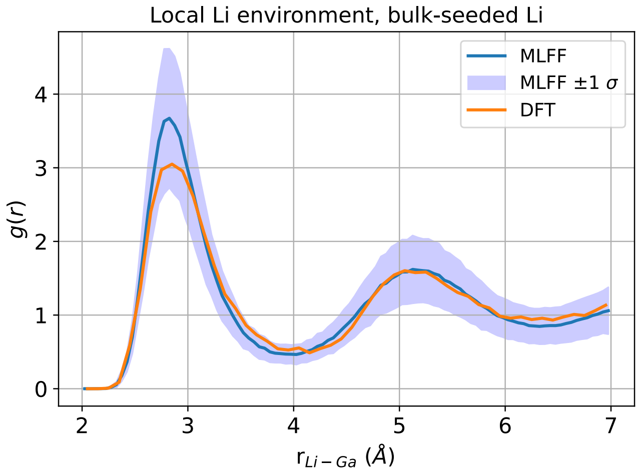

(f) Bulk-seeded Li

Figure S4: Pair distribution functions of X–Ga for all bulk-seeded dopants from DFT (red lines) and MLFF (blue lines) simulations with double-vacuum.

S4 $\bar{z}(t)$ results

S4.1 Main simulations (one oxide interface, one vacuum interface)

Here, we present only the figures not given in the main text.

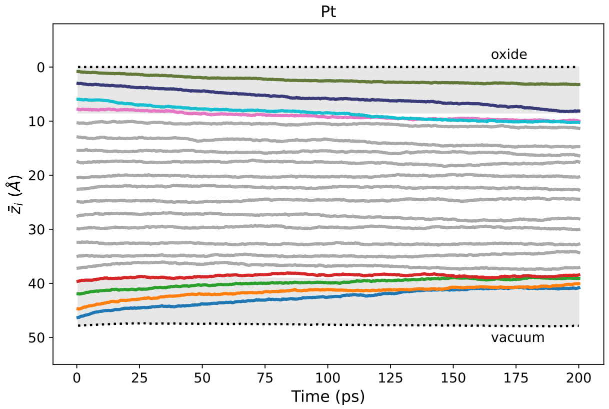

(a) Pt, oxide-vacuum

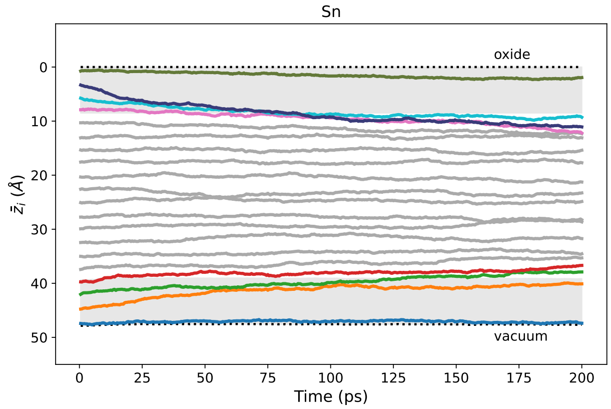

(b) Sn, oxide-vacuum

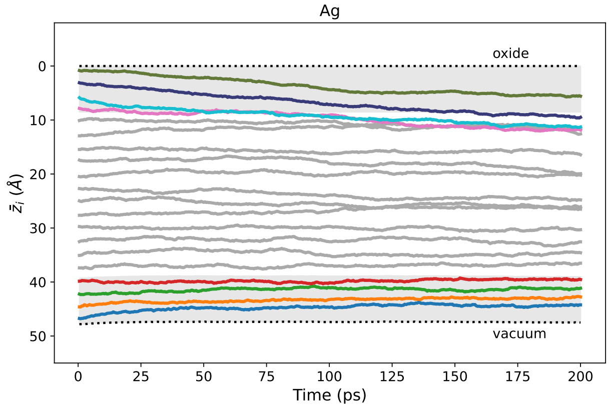

(c) Ag, oxide-vacuum

Figure S5: The $\bar{z}(t)$ is given for each of the 20 dopants for simulations of Pt, Sn and Ag that include one oxide interface and one vacuum interface. The $\bar{z}(t)$ results for other dopant types are presented in the main text.

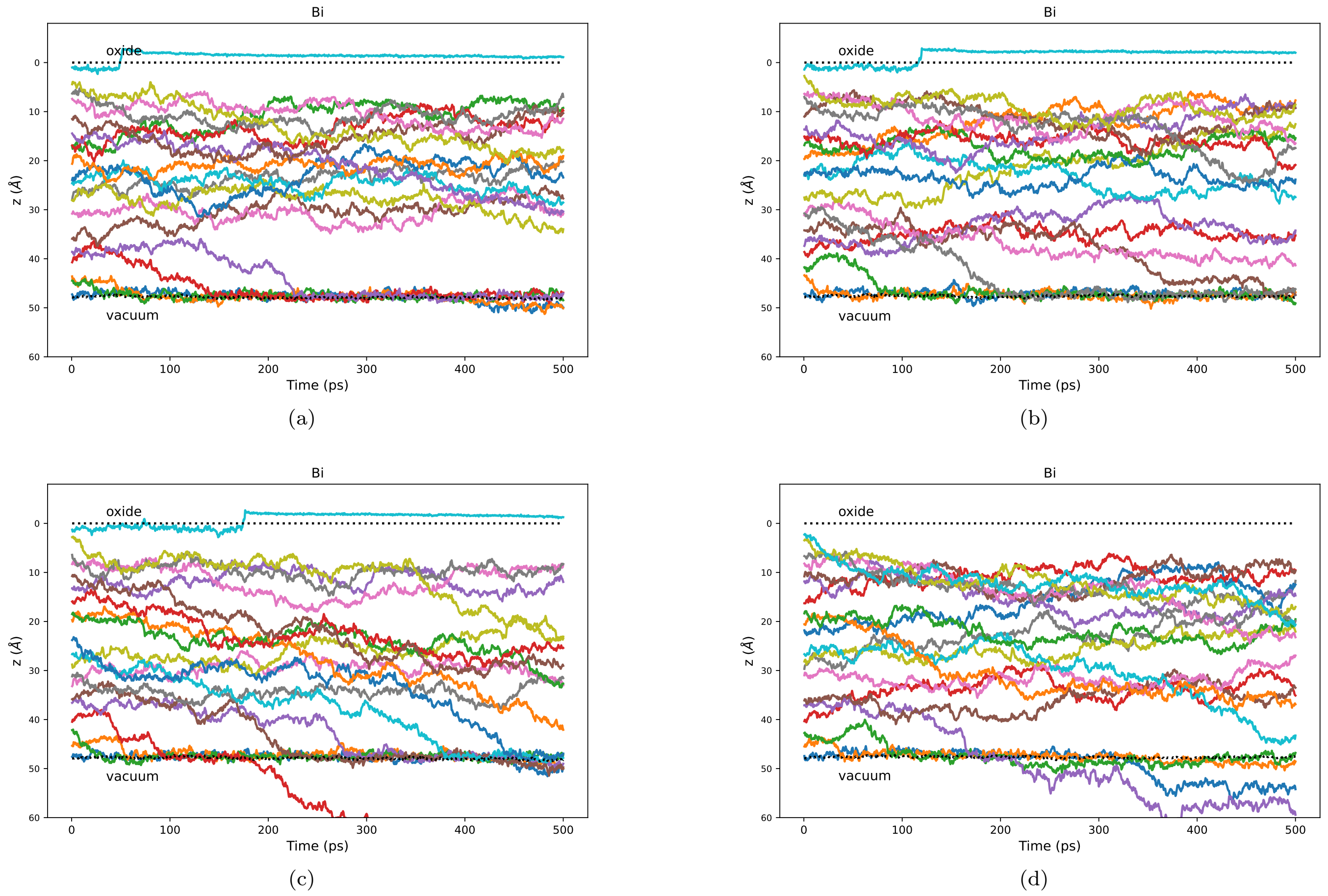

Figure S6: The $z(t)$ is shown for each of the 20 dopants in 4 individual Bi-doped simulations which have been extended by 300 ps for a total duration of 0.5 ns. In all simulations, we observe additional atoms moving towards the vacuum interface. For the simulation shown in (c), in addition to the 3 dopants seeded into the surface layers nearest the oxide, an additional 6 Bi dopants migrate strongly towards the vacuum. For the dopants in (c) and (d) that migrate from the liquid into the vacuum, we note that once Bi atoms move more than ~2.5 Å from the surface, the MLFF becomes unreliable, as it was not trained for such configurations.

S4.2 Double-vacuum control simulations

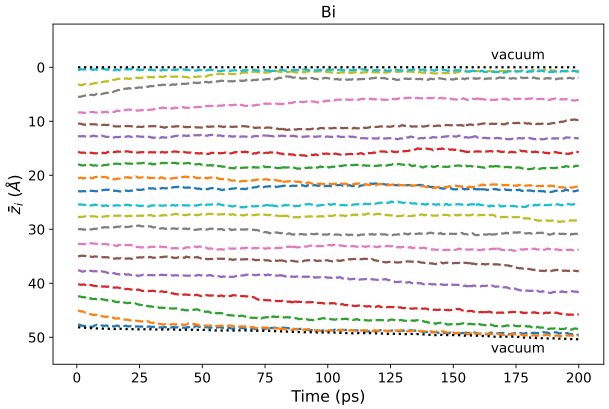

(a) Bi, double-vacuum

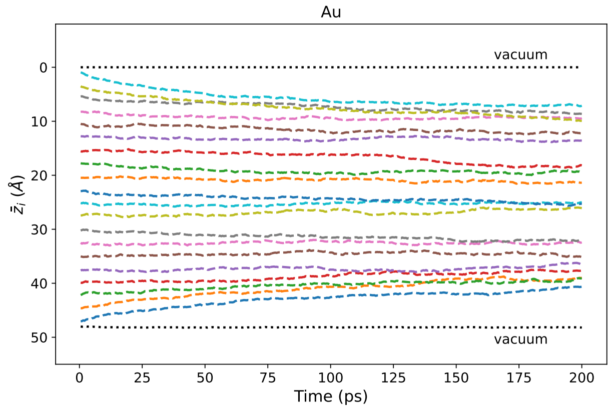

(b) Au, double-vacuum

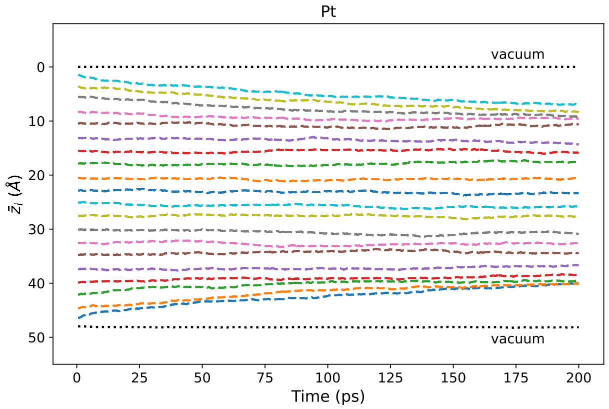

(c) Pt, double-vacuum

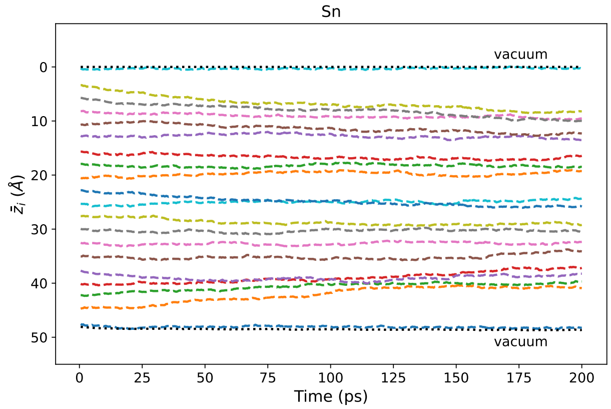

(d) Sn, double-vacuum

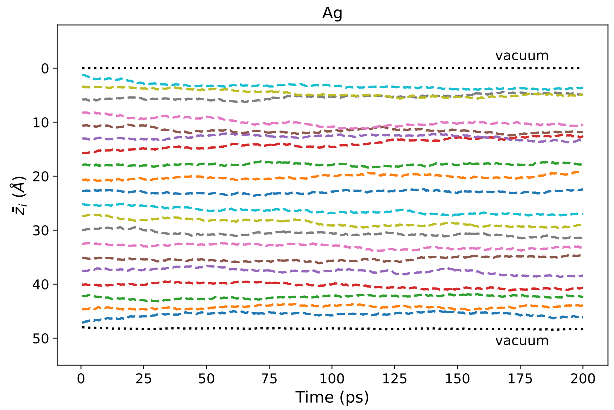

(e) Ag, double-vacuum

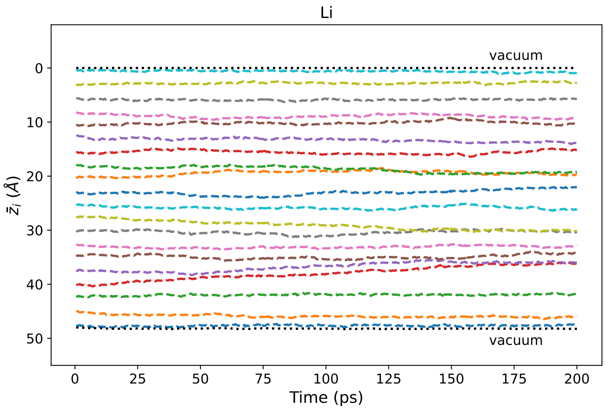

(f) Li, double-vacuum

Figure S7: The $\bar{z}(t)$ is given for each of the 20 dopants for the double-vacuum control simulations of all dopant types.

S5 Diffusion

S5.1 Directional diffusion

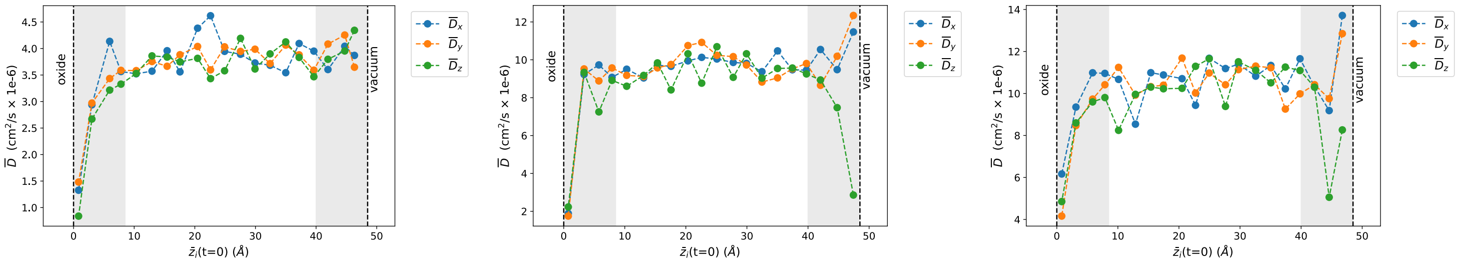

(a) Pt (b) Sn (c) Ag

Figure S8: The average directional diffusion as a function of the average initial z-position, $\bar{z}(t = 0)$, for the Pt, Sn and Ag. Here, the diffusion coefficient is calculated in the Cartesian x, y and z directions for each of the 20 dopant atoms (as described in Section S2.1) plotted as a function of the seeded z-coordinate for each dopant, averaged over all 30 simulations. For reference, the dashed vertical lines show the approximate locations of the interfaces. Note different y-scale for each of the plots, particularly Pt. These directional diffusion plots for Bi, Au and Li are given in the main text.

S5.2 Total diffusion

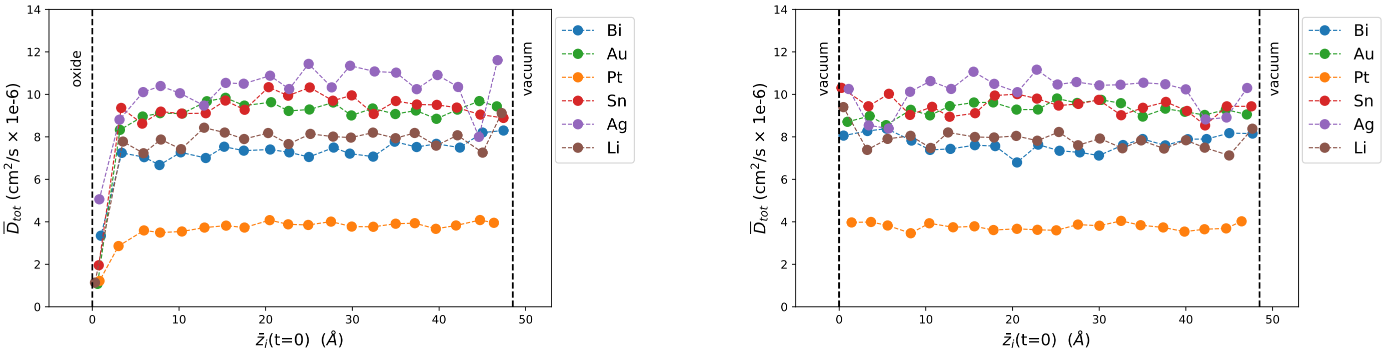

(a) (b)

Figure S9: The $\overline{D}_{tot}$ is given for all 20 dopants as a function of the average initial z-position, $\bar{z}_i(t = 0)$. The total diffusion is calculated as described in Section S2.1, then averaged over all 30 simulations. For reference, the dashed vertical lines show the approximate locations of both interfaces.

S6 Top-surface control simulations

S6.1 $\bar{z}(t)$ results

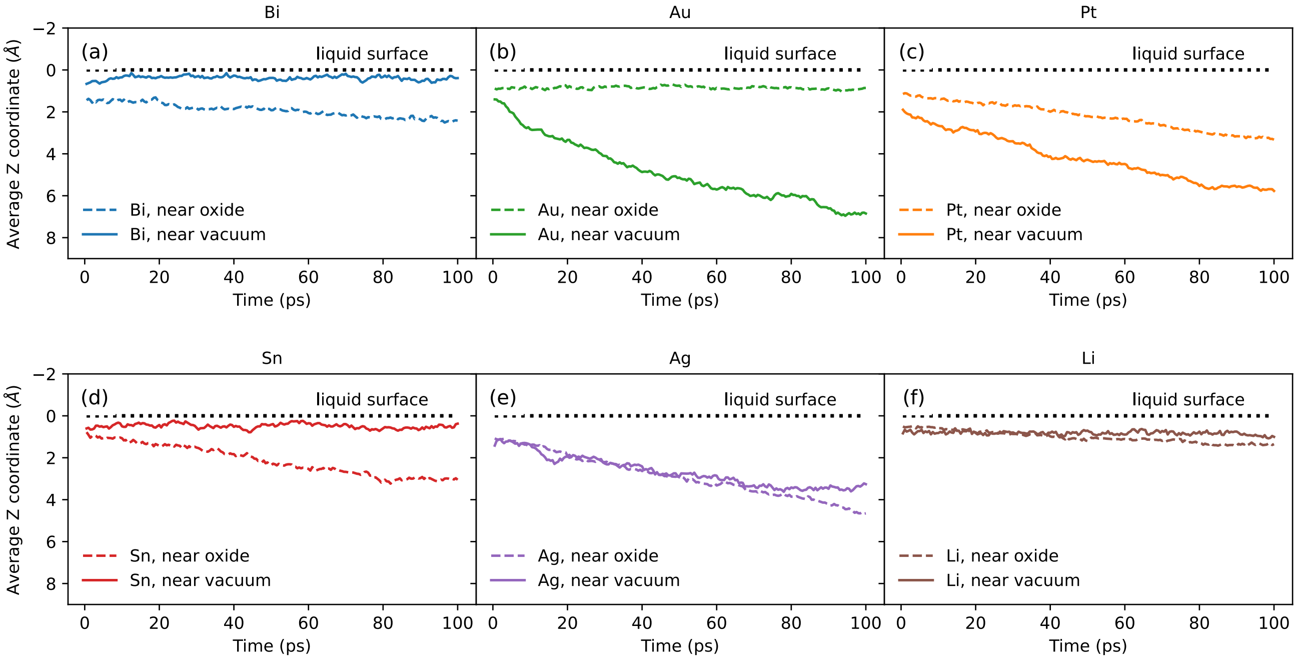

Figure S10: The $\bar{z}(t)$ results for the low dopant ratio (0.055 at.%) simulations, for the dopant seeded into the top-surface gallium liquid layer ($z(t = 0)$ <3 Å). (Dashed lines) indicate results for the dopants seeded in the top liquid layer near the oxide interface, with the dotted-black line indicating the liquid-oxide interface. (Solid lines) indicate results for the dopants seeded in the top liquid layer near the vacuum interface, where the dotted-black line indicates liquid-vacuum interface.

S7 Optimised PBEsol Ga-X dimer energy

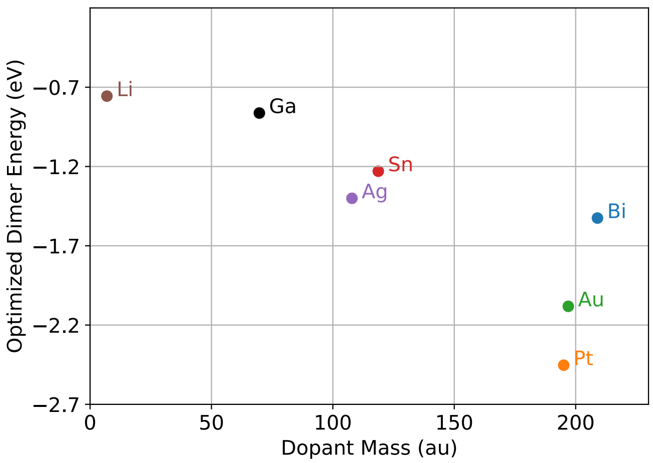

Figure S11: The minimum of the X-Ga dimer interaction energy, where X are the dopants, calculated with the PBEsol functional, plotted as a function of dopant mass. The Ga-Ga minimum dimer interaction energy is also included for reference. Pt has the strongest interaction with gallium of the tested dopants, while Sn is the weakest.

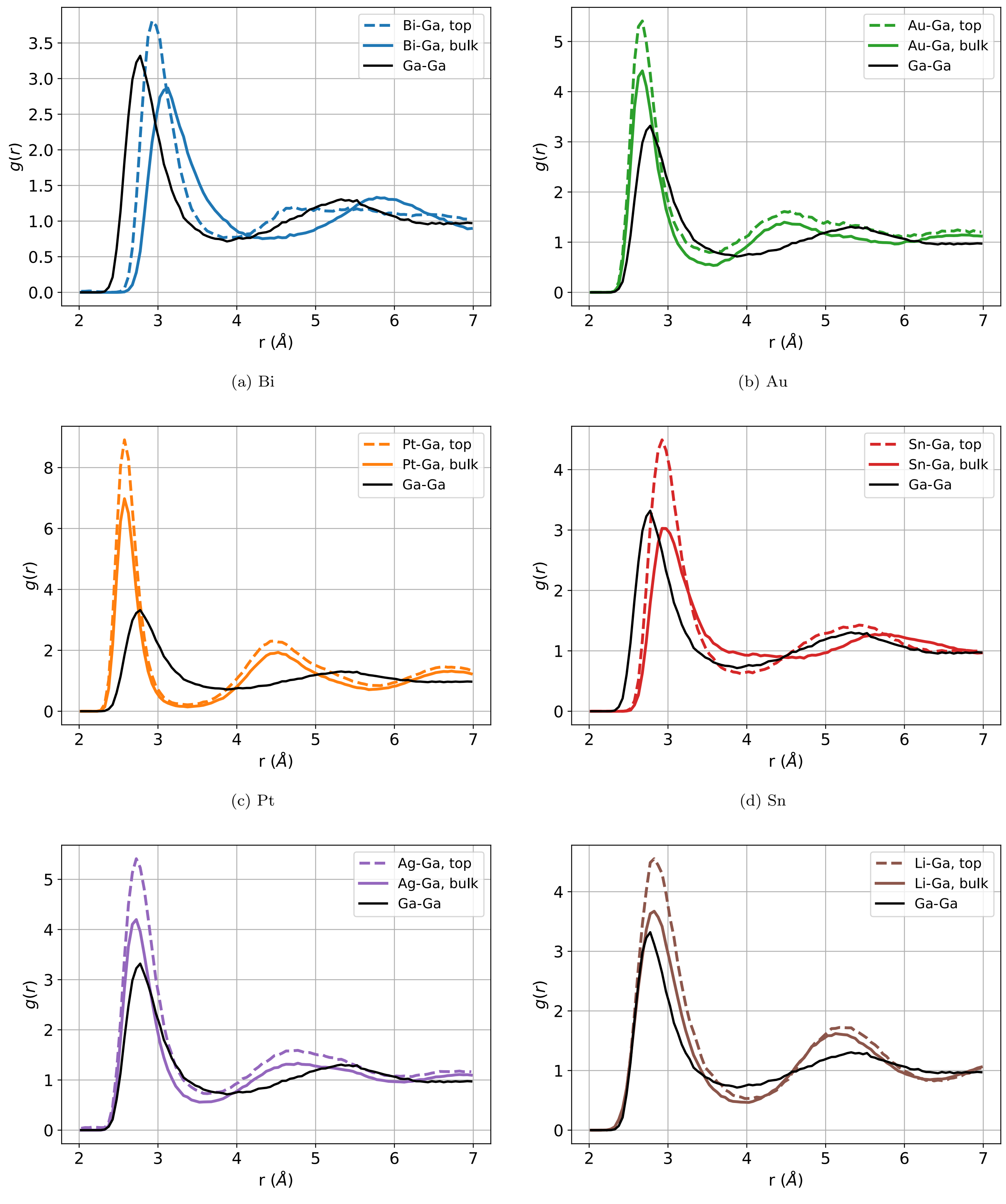

(a) Bi

(b) Au

(c) Pt

(d) Sn

(e) Ag

(f) Li

Figure S12: Pair distribution functions, $g(r)$, for X–Ga compared to Ga-Ga from the low dopant ratio (0.055 at.%) simulations with the one dopant seeded into the top-surface liquid layer: (Dashed, coloured lines) all top-seeded dopants; and (solid, coloured lines) all bulk-seeded dopants. The Ga-Ga $g(r)$ (solid black lines) was calculated for a bulk liquid gallium atom. Note that for top-seeded dopants (dashed lines), the $g(r)$ shell-volume normalisation is based on half the full spherical shell volume, reflecting the fact that atoms at the vacuum interface experience approximately half of a coordination shell due to the absence of neighbouring atoms in the vacuum.
